# Face Masks Impact Auditory and Audiovisual Consonant Recognition in Children With and Without Hearing Loss

**DOI:** 10.3389/fpsyg.2022.874345

**Published:** 2022-05-13

**Authors:** Kaylah Lalonde, Emily Buss, Margaret K. Miller, Lori J. Leibold

**Affiliations:** ^1^Audiovisual Speech Processing Laboratory, Boys Town National Research Hospital, Center for Hearing Research, Omaha, NE, United States; ^2^Speech Perception and Auditory Research at Carolina Laboratory, Department of Otolaryngology Head and Neck Surgery, University of North Carolina School of Medicine, Chapel Hill, NC, United States; ^3^Human Auditory Development Laboratory, Boys Town National Research Hospital, Center for Hearing Research, Omaha, NE, United States

**Keywords:** COVID-19, face mask, hearing loss, children, speech intelligibility, audiovisual perception

## Abstract

Teachers and students are wearing face masks in many classrooms to limit the spread of the coronavirus. Face masks disrupt speech understanding by concealing lip-reading cues and reducing transmission of high-frequency acoustic speech content. Transparent masks provide greater access to visual speech cues than opaque masks but tend to cause greater acoustic attenuation. This study examined the effects of four types of face masks on auditory-only and audiovisual speech recognition in 18 children with bilateral hearing loss, 16 children with normal hearing, and 38 adults with normal hearing tested in their homes, as well as 15 adults with normal hearing tested in the laboratory. Stimuli simulated the acoustic attenuation and visual obstruction caused by four different face masks: hospital, fabric, and two transparent masks. Participants tested in their homes completed auditory-only and audiovisual consonant recognition tests with speech-spectrum noise at 0 dB SNR. Adults tested in the lab completed the same tests at 0 and/or −10 dB SNR. A subset of participants from each group completed a visual-only consonant recognition test with no mask. Consonant recognition accuracy and transmission of three phonetic features (place of articulation, manner of articulation, and voicing) were analyzed using linear mixed-effects models. Children with hearing loss identified consonants less accurately than children with normal hearing and adults with normal hearing tested at 0 dB SNR. However, all the groups were similarly impacted by face masks. Under auditory-only conditions, results were consistent with the pattern of high-frequency acoustic attenuation; hospital masks had the least impact on performance. Under audiovisual conditions, transparent masks had less impact on performance than opaque masks. High-frequency attenuation and visual obstruction had the greatest impact on place perception. The latter finding was consistent with the visual-only feature transmission data. These results suggest that the combination of noise and face masks negatively impacts speech understanding in children. The best mask for promoting speech understanding in noisy environments depend on whether visual cues will be accessible: hospital masks are best under auditory-only conditions, but well-fit transparent masks are best when listeners have a clear, consistent view of the talker’s face.

## Introduction

Face masks are important for limiting the spread of the coronavirus disease 2019 (COVID-19), because they decrease aerosol transmission of the virus during speaking and coughing (Centers for Disease Control and Prevention, 2021a). To limit the spread of the coronavirus, the Centers for Disease Control and Prevention (CDC) stated in the summer of 2020 that individuals aged 2 years and older should wear masks in public and when around people who do not live in their household (Centers for Disease Control and Prevention, 2021b). Many United States schools returned to in-person or hybrid learning for the 2020–2021 school year, with teachers and students wearing face masks in most classrooms. Even after resolution of the current pandemic, face masks may be required to control outbreaks of COVID-19 or other contagious diseases.

While face masks are critically important for preventing the spread of COVID-19, behavioral studies have demonstrated a detrimental effect of face masks on adults’ speech recognition and recall (Wittum et al., 2013; Atcherson et al., 2017; Bottalico et al., 2020; Brown et al., 2021; Magee et al., 2021; Muzzi et al., 2021; Smiljanic et al., 2021; Toscano and Toscano, 2021; Truong et al., 2021; Vos et al., 2021; Yi et al., 2021). Specifically, the use of face masks can lead to difficulties with speech understanding by concealing lip-reading cues and reducing the transmission of high-frequency speech content (Palmiero et al., 2016; Atcherson et al., 2020; Corey et al., 2020; Goldin et al., 2020; Jeong et al., 2020; Pörschmann et al., 2020; Magee et al., 2021; Muzzi et al., 2021; Smiljanic et al., 2021; Toscano and Toscano, 2021; Truong et al., 2021; Vos et al., 2021; Yi et al., 2021). Adults with and without hearing loss report that both factors degrade speech recognition (Naylor et al., 2020; Saunders et al., 2020). When a conversation partner wears a mask, it affects the listener’s hearing and feeling of engagement with the talker (Saunders et al., 2020); among adults, those with hearing loss are more greatly impacted (Saunders et al., 2020).

Most prior studies investigating the effects of face masks on speech understanding are limited to adult subjects. Therefore, effects of face masks on children’s auditory and audiovisual speech understanding in environments, such as classrooms, are not yet understood. Additionally, there are many types of face masks in use, with different effects on acoustic and visual speech cues (e.g., Corey et al., 2020; Yi et al., 2021). The purpose of this study is to examine the effects of various types of face masks on auditory and audiovisual speech recognition in children with and without hearing loss, in hopes of providing recommendations regarding types of face masks that best support children’s speech understanding.

For the general public, including teachers, the CDC recommended non-medical disposable masks, breathable cloth masks made of multiple layers of tightly woven fabrics (such as cotton and cotton blends), or respirators without vents (Centers for Disease Control and Prevention, 2022a). However, teachers and others who interact with individuals who are deaf and hard of hearing were encouraged to wear transparent masks (Centers for Disease Control and Prevention, 2022b). In earlier guidelines, teachers were also encouraged to wear a transparent mask if they interact with students with special education or healthcare needs, teach young students who are learning to read, teach English as a second language, or teach students with disabilities, including hearing loss (Centers for Disease Control and Prevention, 2020). Acoustic attenuation is related to material attributes, such as thickness, weight, weave density, and porosity (Llamas et al., 2008). Among the common alternatives, hospital masks cause the least transmission loss (Atcherson et al., 2020; Bottalico et al., 2020; Corey et al., 2020; Goldin et al., 2020; Vos et al., 2021). Cloth masks vary considerably depending on fabric attributes and number of layers, with one study showing between 0.4 to 9 dB greater attenuation between 2 and 16 kHz for cloth masks than for a disposable hospital mask (Corey et al., 2020).

Opaque face masks conceal a substantial portion of visual cues used for lip-reading and audiovisual speech enhancement. In one study, occluding the lower half of the face decreased adults’ lip-reading of consonant-vowel (CV) syllables from an 80 to 20% accuracy, and their audiovisual enhancement from a ∼21 to 4% benefit (Jordan and Thomas, 2011). Transparent masks (made of plastic or vinyl) provide access to more visual speech cues compared to opaque masks. Adults with moderate to profound hearing loss benefit from visual cues available when the talker is wearing a mask with a transparent window (Atcherson et al., 2017). When listening to speech in noisy backgrounds, adults with normal hearing (ANH) also benefit from visual cues available when the talker is wearing a transparent mask (Yi et al., 2021). However, transparent masks also cause 7–16 dB greater high-frequency attenuation than opaque disposable masks (Atcherson et al., 2020; Corey et al., 2020).

**FIGURE 1:**
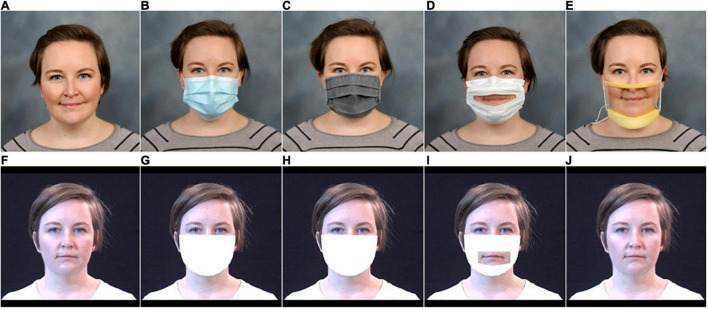
Illustration of the five mask conditions. The top row contains photographs of the five conditions: **(A)** no mask, **(B)** hospital mask, **(C)** fabric mask, **(D)** Communicator™, and **(E)** ClearMask™. The bottom row shows the associated visual face mask simulations: **(F)** no mask, **(G)** hospital mask, **(H)** fabric mask, **(I)** Communicator™, and **(J)** ClearMask™.

Different types of transparent masks vary with respect to visual cues they provide access to. Some transparent masks, such as ClearMask™ (Clear Mask, LLC), conceal very little of the face (Figure 1E). Others, such as The Communicator™ (Safe’N’Clear, Inc.), are primarily opaque with a transparent window in the region of the mouth (Figure 1D). Although not previously examined, differences in visual cues available with different transparent masks might affect audiovisual speech perception. Some non-oral regions of the face contain subtle visual cues that could contribute to audiovisual enhancement (Scheinberg, 1980; Preminger et al., 1998). However, the impact of these non-oral visual cues may be negligible when oral cues are available, because the shape and movement of the oral area are highly correlated with movements of the jaw and cheeks (Munhall and Vatikiotis-Bateson, 1998).

Several recent studies have presented data on the effects of face masks on speech recognition and recall in adults. Among ANH, researchers have reported 12–23% poorer auditory-only (Wittum et al., 2013; Bottalico et al., 2020; Muzzi et al., 2021; Yi et al., 2021) and audiovisual (Brown et al., 2021; Smiljanic et al., 2021; Yi et al., 2021) word and sentence recognition in noise with face masks than without face masks. ANH are worse at recalling sentences presented audiovisually with a cloth or a hospital face mask than without a face mask (Smiljanic et al., 2021; Truong et al., 2021), and they report greater listening effort for speech produced with a face mask than without one (Bottalico et al., 2020; Brown et al., 2021). Among adult hearing aid users with moderate hearing loss, Atcherson et al. (2017) observed 7–8% worse auditory sentence recognition in multi-talker babble with the talker wearing a hospital mask than without one. Among adult cochlear implant users, Vos et al. (2021) observed 26% lower auditory sentence recognition accuracy in quiet with an N95 respirator in combination with a face shield but no effect of the N95 respirator alone. Deficits depend on the amount of background noise in the listening environment (Brown et al., 2021; Smiljanic et al., 2021), with limited effects among ANH tested in quiet or at high SNRs (+5 to +13 dB) (Llamas et al., 2008; Mendel et al., 2008; Atcherson et al., 2017; Brown et al., 2021; Smiljanic et al., 2021; Toscano and Toscano, 2021). Additionally, auditory-only performance may not be affected by face masks in individuals with severe to profound hearing loss (Atcherson et al., 2017; Vos et al., 2021), potentially because their hearing loss restricts the speech bandwidth with and without a face mask.

Recent studies have demonstrated that acoustic effects of face masks significantly impact auditory-only speech recognition in children with normal hearing (CNH; Flaherty, 2021; Sfakianaki et al., 2021), with a similar impact on children and adults. Flaherty, 2021 tested auditory-only speech recognition in a two-talker speech masker among 24 adults and 30 children (8–12 years of age), comparing masked speech recognition thresholds at baseline to a primarily transparent mask (ClearMask™), a face shield, an N95, and a hospital mask. Subjects required a more favorable target-to-masker ratio to understand speech produced with the ClearMask™ and face shield than with no mask, with similar effect sizes in children and adults. Sfakianaki et al. (2021) tested 10 adults and 10 children (6–8 years of age) in quiet, classroom noise, and a two-talker masker, comparing accuracy with no mask to accuracy with a hospital mask. Subjects accurately repeated back fewer words when listening to speech with the hospital mask than without it. There was a non-significant trend for greater impact of the mask in children than in adults.

Although the CDC recommended using a transparent mask when communicating with individuals who are deaf or hard of hearing (Centers for Disease Control and Prevention, 2022b), only one study has examined the effects of face masks on speech perception in children with hearing loss (CHL). Lipps et al. (2021) examined audiovisual word identification in quiet in 13 CHL (3–7 years of age), comparing accuracy at baseline to accuracy with a hospital mask, ClearMask™, and a transparent apron mask. The hospital mask and the transparent apron mask negatively impacted children’s audiovisual word identification accuracy, but the ClearMask™ did not. This finding indicates that a transparent mask is likely the best choice for young CHL listening to speech under quiet audiovisual conditions. We have only begun to scratch the surface of understanding how face masks affect communication in children with and without hearing loss, which limits our ability to make evidence-based recommendations about what type of face mask best supports communication in children with and without hearing loss.

Measurements of the acoustic transmission loss associated with the use of opaque and transparent face masks indicate a trade-off between concealment of visual cues and the degree of high-frequency acoustic attenuation (Atcherson et al., 2020; Corey et al., 2020; Vos et al., 2021). When listening to speech in moderate levels of background noise (−5 dB SNR), the availability of visual cues outweighs the additional high-frequency acoustic attenuation caused by the transparent mask in ANH (Yi et al., 2021). We expect that age- and hearing-related differences in susceptibility to both high-frequency acoustic attenuation and reliance on visual speech cues will affect where children fall in this trade-off. For instance, children may be particularly affected by the acoustic attenuation caused by face masks, because they require greater bandwidths than adults for masked speech understanding (Stelmachowicz et al., 2000, 2001; Mlot et al., 2010; McCreery and Stelmachowicz, 2011). However, the acoustic attenuation of face masks may have a smaller effect on CHL who have poor high-frequency aided audibility, because the hearing loss restricts the speech bandwidth both with and without a face mask. The loss of visual cues resulting from the use of opaque face masks is likely to affect both CNH and CHL, as both groups benefit significantly from visual speech cues (see reviews by Lalonde and McCreery, 2020; Lalonde and Werner, 2021). However, CNH are likely to be less affected than adults by the loss of these visual cues, because they benefit less from visual speech (Wightman et al., 2006; Ross et al., 2011; Lalonde and Holt, 2016). Finally, CHL (and particularly those with more severe hearing loss) may be more impacted by the loss of visual cues, because they benefit more from visual speech than CNH and CHL with less severe hearing loss (Lalonde and McCreery, 2020).

Previous studies examining the impact of face masks on speech understanding have measured effects at the word or sentence level. Face masks likely have a greater detrimental impact on some speech features than others. Reduced transmission of high-frequency speech content is likely to degrade the perception of speech features based primarily on high-frequency acoustic cues, such as consonants and specifically their place of articulation (Stevens, 2000). Similarly, reduced transmission of visual speech cues is likely to degrade the perception of speech features that are easy to speech-read, such as consonant place of articulation (Binnie et al., 1974; Owens and Blazek, 1985).

The purpose of this study was twofold. First, we aimed to determine the impact of face masks on auditory and audiovisual speech recognition in noise among CNH and CHL. Second, we aimed to determine what type of face mask may best support communication in these groups. We used four types of face masks: a hospital mask, a fabric mask, a primarily transparent mask (ClearMask™), and a primarily opaque mask with a transparent window (The Communicator™). We compared performance with these face masks to baseline conditions with no mask. Audiovisual conditions were included to investigate the impact of face masks on communication under ideal conditions. Auditory-only conditions were included to evaluate performance under less-than-ideal conditions, based on limited evidence that young children do not consistently orient to the target talker the way adults do (Ricketts and Galster, 2008).

Based on previous studies and children’s susceptibility to the detrimental effects of decreased auditory bandwidth, we expected all the masks to affect auditory-only performance in children with good high-frequency aided audibility, including CNH and some CHL. More specifically, we expected that reduced transmission of high-frequency speech content would affect the discrimination of consonants, especially consonant place of articulation (Stevens, 2000). Based on acoustic transmission data in previous studies, we expected hospital masks to affect auditory-only performance the least and transparent masks to affect auditory-only performance the most. In children with poor high-frequency aided audibility, we expected a smaller difference between scores in the auditory-only conditions. In audiovisual conditions, we expected opaque masks to reduce the transmission of visual speech cues, which would affect the discrimination of consonants, especially consonant place of articulation (Binnie et al., 1974; Owens and Blazek, 1985). As such, under audiovisual conditions, we expected children with poor high-frequency aided audibility to perform best with the transparent masks, because they benefit from visual speech cues (Lalonde and McCreery, 2020) and may be less impacted by the high-frequency attenuation caused by the masks. It is uncertain what to predict for CNH and CHL with good high-frequency aided audibility with respect to the trade-off between loss of visual cues and high-frequency attenuation. The results from this study will provide an evidence base for advising educators and others who work with children as to the best face masks for promoting speech understanding.

## Overview, General Materials, and General Methods

This study was designed to test the effects of four types of face masks on auditory and audiovisual speech recognition in noise by ANH, CNH, and CHL. The four types of masks (Figures 1B–E) include a disposable hospital mask, a homemade pleated cloth mask with two layers of cotton blend, the Communicator™, and the ClearMask™. The Communicator™ is an FDA-registered single-use, disposable device that meets ASTM Level 1 hospital mask standards and includes a fog-resistant transparent window. The ClearMask™ is an FDA-approved, class II single-use transparent face mask that meets ASTM Level 3 standards. It serves the same function as traditional masks and provides a full, anti-fog plastic barrier.

We simulated the effects of face masks on audiovisual stimuli by filtering the acoustic speech from an unmasked talker to match the long-term average spectrum of each mask and by overlaying mask shapes onto videos of the unmasked talker. This method does not capture differences in production that talkers may adopt when wearing a face mask, but it has the advantage of ensuring that idiosyncrasies in production across conditions do not affect results.

Experiment 1 was conducted by delivering research equipment to the homes of CHL. CHL and members of their household completed auditory-only and audiovisual speech recognition tests in noise at 0 dB SNR. In Experiment 2, young ANH completed the same test in a laboratory sound booth. The effects of face masks vary depending on baseline difficulty; mask effects differ in subjects as a function of SNR (Toscano and Toscano, 2021). Therefore, the ANH in Experiment 2 were also tested at −10 dB SNR to better match the performance level of the CHL tested in Experiment 1. Both experiments were reviewed and approved by the Boys Town National Research Hospital Institutional Review Board. Adult participants provided their written informed consent to participate in the study; child participants and their caregivers provided written assent and permission, respectively.

### Stimuli

Target stimuli included 36 audiovisual recordings of a 32-year-old female native speaker of mainstream American English (author KL) repeating CV words with the vowel /i/ and the format, “Choose /CV/”. The 12 CVs (/bi/, /si/, /di/, /hi/, /ki/, /mi/, /ni/, /pi/, /∫i/, /ti/, /vi/, and /zi/) were the same CVs used in a previous study evaluating auditory-only speech perception in children (Leibold and Buss, 2013), based on the Audiovisual Feature Test for Young Children (Tyler et al., 1991). Three tokens of each CV were used so that idiosyncratic differences between the videos (e.g., blinking) could not be used to discriminate the syllables. Including the carrier, these recordings had a mean duration of 856 ms (728–941 ms) and a mean F0 of 238 Hz (212–276 Hz).

The original unmasked target stimuli were professionally recorded in a large sound-attenuating booth with a Sennheiser EW 112-P G3-G lapel microphone and a JVC GY-GM710U video camera. Final Cut Pro video editing software was used to splice the recordings into individual videos, which began 333 ms (10 frames) before the onset of acoustic speech and ended 333 ms after the end of the acoustic speech, with the exception that additional frames may have been added to avoid beginning or ending the video mid-blink. Using Adobe Audition, the intensity of each sound file was adjusted so that the total RMS of the speech portion of all the files matched.

#### Acoustic Mask Simulation

**FIGURE 2:**
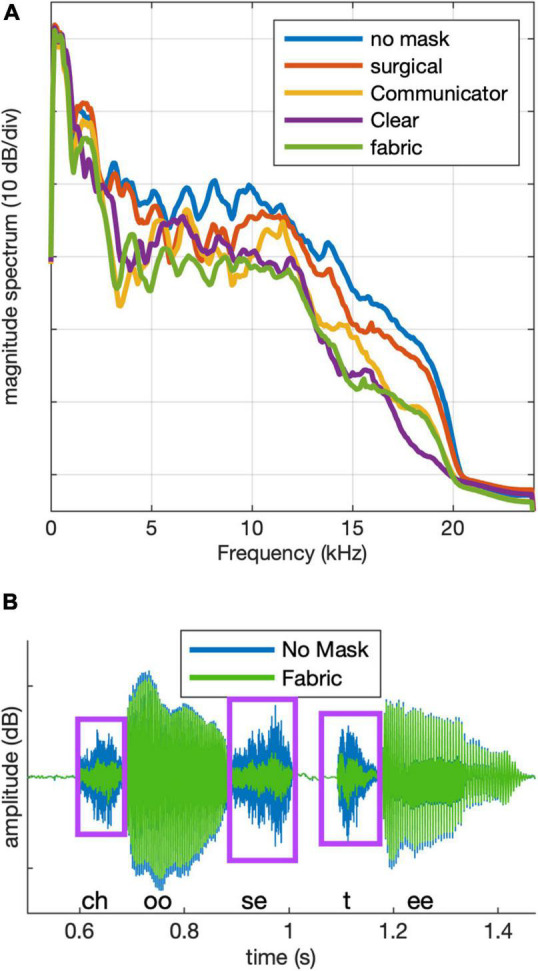
**(A)** Long-term magnitude spectra for stimuli in each of the five conditions. **(B)** Example target stimulus waveform after filtering for the no mask (blue) and fabric mask (green) conditions. Boxes indicate consonant regions.

Target stimuli in the four mask conditions were generated by filtering the original (no mask) target recordings. Filters were constructed based on recordings of the rainbow passage (Fairbanks, 1969), produced by the target talker, each lasting 1.7–1.9 min. Two recordings were made using a Shure-KSM42 (condenser) microphone in each of five conditions: no mask, hospital mask, fabric mask, ClearMask™, and Communicator™. The two recordings for each condition were concatenated, and the result was transformed into the frequency domain using the *pwelch* function in MATLAB (Mathworks), with a 512-point window and a 256-point overlap between sequential windows. Attenuation as a function of frequency for each mask was determined as the difference in amplitude spectrum relative to the no-mask condition. These attenuation functions were used to generate 128-point FIR filters using the *fir2* function in MATLAB. An all-pass filter was generated for use with the no-mask stimuli; this was done so the stimulus preparation steps were identical across stimuli. The amplitude spectrum of the no-mask recordings was also used to construct a filter for generating speech-shaped noise, following the same procedures. Stimuli were filtered using the *filter* function in MATLAB and saved to a disk. Long-term average magnitude spectra for targets in each condition are shown in Figure 2A. The speech-shaped noise was 30 s in duration, and its long-term average magnitude spectrum (not shown) was similar to that of the no-mask targets.

As illustrated in Figure 2A, power spectra in the four mask conditions were similar to the power spectrum of the no-mask to within approximately ±6 dB up to 2 kHz. Mean attenuation between 2 and 16 kHz was 2.4 dB for the hospital mask, 5.5 dB for the Communicator™, 5.9 dB for the ClearMask™, and 8.2 dB for the fabric mask. Peak attenuation within a 1/3-octave-wide band by mask type was 5.5 dB at 8 kHz for the hospital mask, 14.9 dB at 3.6 kHz for the Communicator™, 11.9 dB at 16 kHz for the ClearMask™, and 12.7 dB at 3.2 kHz for the fabric mask. Figure 2B shows an example stimulus waveform after filtering for no mask (blue) and fabric mask (green) conditions. The mask causes greater attenuation of the consonants than the vowels. We observed similar acoustic differences between the mask and no mask conditions in recordings of the talker wearing a mask.

#### Visual Mask Simulation

To visually simulate face masks, video files were processed using specialized software that automatically determines the position of 66 points on the face in each frame of the videos (Saragih et al., 2011; available at osf.io/g9jtr/). These included 17 points on the perimeter of the lower half of the face, 18 points at the inner and outer rim of the lips, 6 points for each eye, 5 points for each eyebrow, 4 points on the bridge of the noise, and 5 points at the bottom of the nostrils.

The *x* and *y* coordinates of these points were imported into MATLAB. The Computer Vision Toolbox was used to superimpose filled white polygons onto each video frame. For opaque face masks (hospital, cloth), the size and shape of the polygon were created based on the location of the 17 points at the perimeter of the lower half of the face and the marker at the upper middle of the bridge of the nose. An example of the simulated opaque face mask is shown in Figure 1G. The simulated Communicator™ mask was created in a similar fashion, except that two filled polygons were used to create the effect of a cutout in the middle of the simulated opaque mask shape. Adjustments to the simulated masks were made to correct for problems noted by independent observers (see Supplementary Material). An example of the simulated Communicator™ mask is shown in Figure 1I. For the ClearMask™, no mask was superimposed. The stimuli are available at https://osf.io/5wapg.

## Experiment 1

The first experiment was conducted remotely in participants’ homes. The goal was to evaluate auditory-only and audiovisual speech recognition in the five conditions. Remote data collection made it possible to collect data without bringing the subjects to the laboratory. Collecting data from members of the CHL’s household increased the efficiency of the protocol and reduced the variance of test conditions across participant groups.

### Materials and Methods

#### Participants

**FIGURE 3:**
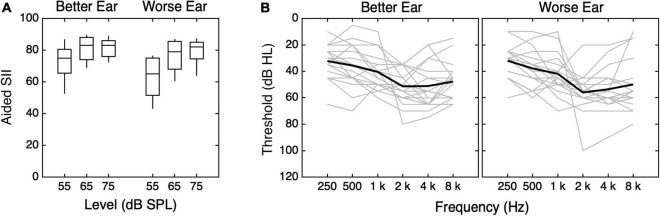
Values of **(A)** SII and **(B)** pure-tone thresholds for children with hearing loss (CHL). **(A)** The distribution of SII is plotted as a function of stimulus level, with results shown separately for the better and worse ears, as indicated at the top of the panel. Horizontal lines indicate the median, boxes span the 25th to 75th percentiles, and vertical lines span the 10th to 90th percentiles. **(B)** Pure-tone thresholds for the better and worse ears are shown. Better and worse ear were identified for each subject based on SII55. Better-ear thresholds are shown in the left panel, and worse-ear thresholds are shown in the right panel. Individual data are shown in gray, and means are shown with thick black lines.

Eighteen children with bilateral hearing loss (8 males) and 39 members of their households completed a remote study. CHL varied in age, between 7.4 and 18.9 years (mean = 12.7 years, SD = 3 years). Sixteen had sensorineural hearing loss, and two had mixed hearing loss. Audiograms and aided Speech Intelligibility Index (SII) scores at 65 and 75 dB SPL were available from 17 children’s previous research visits, 3–24 months before data were collected for this study (median 7.5 months). An audiogram (but not aided SII scores) was acquired from the other child’s previous clinical visit 7.5 months before data were collected for this study. Mean better-ear aided SII was 72.1 at 55 dB SPL, 80.8 at 65 dB SPL, and 81.2 at 75 dB SPL. The distribution of better-ear aided SII scores and audiograms for each of the CHL are shown in Figure 3. Medical records indicate that 11 children had congenital hearing loss. Five passed their newborn hearing screening and had hearing loss identified between age 4 and 7 years. Two others had hearing loss identified at age 2 or 3, but no newborn hearing screening results were reported. Family members of the CHL included 16 children with parent-reported normal hearing (9 males) between 7.5 and 19.8 years of age (mean = 12.1 years, SD = 3.5 years) and 23 adults with self-reported normal hearing (8 male) between 33.6 and 50.7 years of age (mean = 43 years, SD = 4.3 years). No audiometric data were available for family members of the CHL. Sample sizes are consistent with previous studies involving CHL (e.g., Leibold et al., 2013, 2019; Lalonde and McCreery, 2020).

#### Apparatus

The experiments were conducted using custom software on a 13″ MacBook Air laptop (MacOS Catalina 10.15.6). Auditory stimuli were routed directly from the laptop to two loudspeakers (QSC CP8 compact powered 90°) *via* a breakout cable with dual XLR inserts. The equipment was placed on a mat that was marked to show the intended location of the laptop and speakers (see Supplementary Figure 3). Speakers were placed 23 cm away from the edge of the laptop and oriented toward the participants’ ears. A manual explaining how to set up the equipment and run the experiment was also provided.

#### Task

The participants completed a closed-set consonant identification task in speech-shaped noise using a picture selection response. On each trial, the noise was presented at 70 dB SPL, and the target was presented at 0 dB SNR. The noise had 20-ms ramps at onset and offset. The videos began 186 ms after the initiation of noise onset and ≥333 ms before the onset of speech-related movements, ensuring that each video began with a neutral face. On average, acoustic speech began 756 ms (SD = 11 ms) after the offset of the noise ramp. The participants identified the CV token from a matrix of 12 alphabetically ordered illustrations that appeared immediately after the stimulus (Supplementary Figure 4). The same illustrations and similar methods were used in a previous experiment with children as young as 5 years old (Leibold and Buss, 2013). In each trial, the stimulus was presented once; there was no option to repeat the stimulus. Participants were instructed to take their best guess if they were unsure.

The participants completed testing in 11 randomly ordered conditions (four mask conditions conducted in auditory-only and audiovisual modalities and the no mask condition in auditory-only, audiovisual, and visual-only modalities). The five conditions included a no-mask baseline (Figures 1A,F) and simulations of speech produced with a hospital mask (Figures 1B,G), a fabric mask (Figures 1C,H), and two transparent masks [ClearMask™ (Figures 1E,J) and Communicator™ (Figures 1D,I)]. In audiovisual conditions, the participants saw a synchronous and congruent video of the speaker, with or without a simulated face mask. In auditory-only conditions, the participants saw a blank gray screen during auditory stimulus presentation. The 36 consonant stimuli (12 CVs, 3 samples each) were presented in random order in a block of trials.

#### Procedure

Informed consent was obtained by a research audiologist by videoconferencing *via* the HIPAA-protected WebEx Internet-based application and electronic consent forms developed in the Internet-based software called Research Electronic Data Capture (REDCap). All the participating family members were consented together, and each individually signed their forms electronically. Following completion of all consent paperwork, the audiologist provided an overview of the instructions. The participants were instructed to complete the testing in a quiet space free from distraction and to listen carefully to the woman who appeared on the screen. The audiologist arranged a time to deliver test equipment to the participants’ homes and provided her contact information so that she could be available for troubleshooting.

The participants received a manual with the test equipment. In the manual, parents were instructed to choose the most technologically savvy and most available participant in the home to participate first, to ensure the equipment was set up properly, and that the person could assist all other participants at home if needed. Furthermore, the participants were instructed to set up the equipment in a quiet space, away from all other participants, to avoid premature exposure to the stimuli. The recommended equipment configuration was to place the mat on a long table (at least 1.52 m × 0.76 m). There were cases in which a family member did not have a table large enough on which to set the equipment, so they sat with the equipment on the floor. Once the equipment was in place, parent participants were asked to test each speaker with a feature on the user interface. To ensure appropriate calibration, the participants were instructed to keep the laptop set at full volume. The speakers were also fitted with a custom 3D printed plastic barrier that prevented the participants from adjusting the speaker volume. It was recommended that the equipment stay in place once it was set up.

When they were ready for the test, the participants were instructed to sit directly in front of the laptop. Instructions specified that the CHL should wear their hearing aids in their typical configuration while completing the study. With the help of the manual, the participants were instructed to test themselves in the randomized order indicated on their individualized data collection sheet. Despite these instructions, 15 participants tested conditions in alphabetical order [some participants ran both sets of each condition in alphabetical order (AA, BB, CC, …), while others ran through each condition in alphabetical order twice (ABC…, ABC…)]. This resulted in the following order: no mask, ClearMask™, Communicator™, fabric mask, and hospital mask, with each auditory condition preceding the corresponding audiovisual condition; the visual-only condition was last. There were also instances in which participants began testing and then stopped in the middle of a condition once they realized they were not following the order on their individualized datasheet. These incomplete conditions were excluded from the analysis. The parents were asked to assist their children during testing. Total test time was 1.25 to 2 h. Breaks were suggested for all the participants, but strongly encouraged for younger participants. Most of the participants completed the testing in one sitting; others split up the testing into two sessions. The participants were paid with an electronic gift card for their participation. A research audiologist was on stand-by remotely to answer questions or help with the protocol.

#### Analyses

Analyses were performed in RStudio (version 1.2.1335). Linear mixed-effects models were fitted using the *lmer* and *anova* functions in the *lmerTest* package (Kuznetsova et al., 2017). The *anova* function provided *F*-statistics for the model generated using the *lmer* function. Non-significant interactions were systematically eliminated to arrive at the final model. Reference conditions were systematically varied as needed for *post hoc* comparisons. Auditory-only and audiovisual proportion correct data were transformed into rationalized arcsine units (RAUs) for statistical analyses.

### Results

**FIGURE 4:**
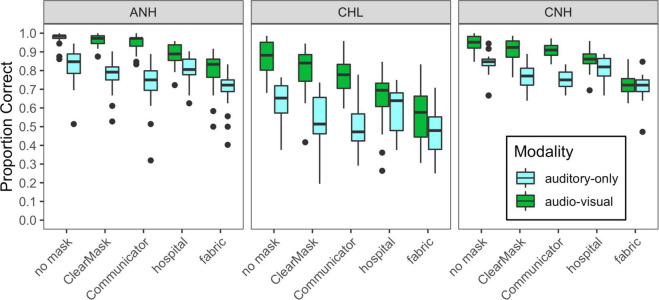
Distribution of scores for each of the auditory-only and audiovisual stimulus conditions, plotted separately for each of the three groups of subjects tested in Experiment 1, as indicated at the top of each panel. Mask type is indicated on the horizontal axis, and cue condition is indicated with box fill, as defined in the legend. Vertical lines indicate the median, boxes span the 25th to 75th percentiles, and vertical lines span the 10th to 90th percentiles.

Most subjects in Experiment 1 completed two runs of data collection in each condition, but there were exceptions. Because of a programming error, two subjects in each group heard the no-mask audiovisual condition at 20 dB SNR rather than 0 dB SNR. These data were omitted. The visual-only condition was added after the data collection commenced; as a result, data in the visual-only condition were only obtained for 16/23 ANH, 11/16 CNH, and 12/18 CHL. Of the remaining 603 cases (subjects × conditions, minus missing data), 27% included data from a single run, 72% from two runs, and only 1% from more than two runs. Figure 4 shows the distribution of mean performance plotted by stimulus condition for the three groups of subjects tested in Experiment 1.

Overall, auditory-only and audiovisual accuracies were poorer and more variable for CHL than for the two groups with normal hearing. These group differences were similar for auditory-only and audiovisual conditions. Audiovisual accuracy was higher than auditory-only accuracy, especially in the no mask, ClearMask™, and Communicator™ conditions. The masks degrade performance relative to the no mask condition, but the impact varied across groups, modalities, and mask conditions.

The initial linear mixed-effects model examining RAU-transformed accuracy included fixed effects and interactions of mask condition, modality, and group, as well as a random intercept per subject. The baseline no mask condition, auditory-only modality, and CNH group served as the reference condition for the model. The final model included significant main effects of modality (*F*_1_,_916_ = 981.9, *p* < 0.0001), mask condition (*F*_4_,_916_ = 176.6, *p* < 0.0001), and group (*F*_2_,_55_ = 37.4, *p* < 0.0001), as well as interactions of modality and mask condition (*F*_4_,_916_ = 72.1, *p* < 0.0001) and modality and group (*F*_2_,_916_ = 18.9, *p* < 0.0001). Model estimates and *post hoc* comparisons are shown in Supplementary Table 1. The CHL had lower speech recognition accuracy than the groups with normal hearing, in both the auditory-only and audiovisual modalities. The ANH and the CNH performed similarly in the auditory-only modality, but the ANH were more accurate than the CNH in the audiovisual modality. All the groups demonstrated significantly better overall performance in the audiovisual modality than in the auditory-only modality. The effect of modality differed across all the groups. The ANH exhibited greater audiovisual benefit (greater difference between the auditory-only and audiovisual modalities) than the CNH and the CHL. The CHL exhibited greater audiovisual benefit than the CNH.

The effect of mask type differed across modalities but not across groups. In the auditory-only modality, the ClearMask™, Communicator™, and fabric masks significantly degraded performance relative to the no-mask condition (*B* ≤ −8.15, *t*_916_ ≤ −6.337, *p* < 0.0001), but the hospital mask did not (*B* = −2.17, *t*_916_ = −1.7, *p* = 0.0895). The masks differed significantly in the severity of degradation. The fabric mask had the greatest detrimental impact on performance, and the hospital mask had the least impact; the Communicator™ mask had a greater detrimental impact than the ClearMask™. This pattern of results is consistent with the degree of high-frequency acoustic attenuation shown in Figure 2. In the audiovisual modality, all four of the masks degraded performance relative to the no-mask condition (*B* ≤ −7.23, *t*_916_ ≤ −5.391, *p* < 0.0001). The opaque masks (hospital and fabric) had a greater detrimental impact than the transparent masks (ClearMask™, Communicator™) due to loss of visual cues. The fabric mask resulted in the greatest degradation in audiovisual conditions. Significant audiovisual benefit was observed for the no mask, Communicator™, and ClearMask™ conditions, and did not differ among these conditions. No significant audiovisual benefit was observed for the hospital and fabric mask conditions with the CNH as the reference group. Although there was no significant three-way interaction, audiovisual benefit was observed for the hospital and fabric mask conditions with the CHL or the ANH as the reference group.

Although this study was not designed to examine individual differences, we explored the impact of child age on the results reported above. Overall, older children recognized speech in noise with greater accuracy than younger children. An initial linear mixed-effects model examining RAU-transformed accuracy included fixed effects of mask condition, modality, group (CNH and CHL), and age; three-way interactions of modality, age, and group and modality, age, and mask condition; and a random intercept per subject. In addition to the effects and interactions of condition, modality, and group noted above, the model indicated a significant effect of age (*F*_1,32_ = 13.5, *p* = 0.0009), an interaction of age and modality (*F*_1,517_ = 5.5, *p* = 0.0193), and a marginal three-way interaction of age, modality, and mask condition (*F*_4,517_ = 2.1, *p* = 0.0802). Age effects did not significantly differ between the CNH and CHL groups. The *post hoc* comparisons demonstrated that age effects were present in every condition (*B* ≥ 1.6, *t*_86_ ≥ 2.648, *p* ≤ 0.0097) except for the auditory-only no mask and auditory-only hospital mask conditions (*B* ≤ 1, *t*_86_ ≤ 1.795, *p* ≤ 0.0762). In auditory-only conditions, younger children were more negatively impacted by the ClearMask™, Communicator™, and fabric mask than older children (*B* ≥ 1.1, *t*_517_ ≤ 2.065, *p* ≤ 0.0395). This could indicate greater bandwidth requirements in younger children, but ceiling effects could produce this pattern of results. These ceiling effects, along with limited representation of older children in our sample (*n* = 6 age 15 and higher), prohibit strong conclusions about how the negative impact of masks varies from 7 to 19 years of age.

## Experiment 2

The second experiment was conducted in a sound booth in the laboratory. The goal of Experiment 2 was twofold. One goal was to replicate the results obtained remotely from the ANH under more controlled conditions. Another goal was to obtain data from adults at a more challenging SNR (−10 dB) to approximately match the overall performance level of CHL tested at 0 dB SNR.

### Materials and Methods

#### Participants

Fifteen ANH (3 males) between 19 and 28 years of age (mean = 22.3 years, SD = 2.4 years) participated. All the participants passed a pure-tone hearing screening bilaterally at 20 dB HL at octave intervals from 0.25 to 8 kHz. They also demonstrated at least 20/30 vision bilaterally, with or without corrective lenses, based on a Snellen eye chart screening. Ten subjects provided data at 0 dB SNR and ten at −10 dB SNR. The first five subjects tested at 0 dB SNR provided pilot data at −8 dB SNR. Five subjects were tested at both 0 dB SNR and −10 dB SNR, and another five subjects were tested only at −10 dB SNR. These sample sizes were determined based on previous studies with similar comparisons to CNH and/or CHL (Leibold and Buss, 2013; Lalonde and McCreery, 2020).

#### Apparatus, Task, and Procedures

The task, apparatus, and procedures were the same as in Experiment 1 but with a few exceptions. The experiment was completed in a laboratory setting. The equipment was set up in a sound booth by an experimenter in the same configuration as in Experiment 1, except that two JBL Professional IRX108BT portable powered loudspeakers (James B. Lansing Sound, Inc.) were used. Written consent was obtained in person by a research assistant who also provided an oral description of the study at the start of the session. The participants were tested in one or two sessions, depending on the number of SNRs in which they completed testing. During each session, the participants completed each of the four masks and no mask conditions in auditory-only and audiovisual conditions twice. In two cases, a participant mistakenly completed a condition three times. During one session (randomized across subjects), the participants also completed the visual-only condition twice. Two participants completed the visual-only condition during both sessions. The order of SNRs and conditions was randomized across the participants. Two subjects completed the conditions in alphabetical order when tested at 0 dB SNR.

### Results

**FIGURE 5:**
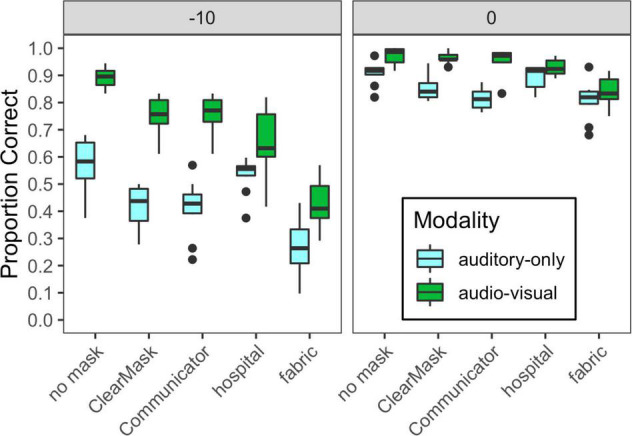
Distribution of scores for each of the auditory-only and audiovisual stimulus conditions, plotted separately for each of the two SNRs tested in Experiment 2, as indicated at the top of each panel. Mask type is indicated on the horizontal axis, and cue condition is indicated with box fill, as defined in the legend. Vertical lines indicate the median, boxes span the 25th to 75th percentiles, and vertical lines span the 10th to 90th percentiles.

Figure 5 shows the distribution of performance for ANH tested in the lab, plotted by stimulus condition. The results for subjects tested at −10 dB and 0 dB SNR are shown in the right and left panels, respectively. A linear mixed-effects model with a random intercept per subject was used to examine the effects of SNR, mask condition, modality, and their interactions on consonant recognition. Model estimates and *post hoc* comparisons are shown in Supplementary Table 2. The final model included significant interactions of SNR and modality (*F*_1_,_372_ = 46.4, *p* < 0.0001), SNR, and mask condition (*F*_4_,_372_ = 13.1, *p* < 0.0001), and modality and mask condition (*F*_4_,_372_ = 25.8, *p* < 0.0001).

The interactions of SNR with modality and SNR with mask condition reflect increases in the effects of mask and modality at −10 dB SNR compared to 0 dB SNR, likely because ceiling effects were eliminated at the more difficult SNR. At −10 dB SNR, auditory-only performance was degraded by all the mask types, with hospital masks having the smallest effect and fabric masks having the largest. At 0 dB SNR, auditory-only performance was degraded by the ClearMask™, Communicator™, and fabric mask, and was poorer for the fabric mask than for the ClearMask™. At both SNRs, audiovisual performance was degraded by all the mask types, with the same pattern as in Experiment 1. At −10 dB SNR, there was a significant audiovisual benefit in all the mask conditions. At 0 dB SNR, audiovisual benefit was significant in all mask conditions except the hospital mask. Audiovisual benefit was greater for the transparent masks and no mask conditions as compared to the opaque masks. The significant interaction of mask and modality reflects reduced audiovisual benefit for the two opaque masks, as observed in Experiment 1.

Comparing the ANH tested in the lab at 0 dB SNR (Figure 5, right) to the ANH tested remotely at the same SNR (Figure 4, left), there was a small but significant overall effect of group (*F*_1_,_31_ = 5.9, *p* = 0.0215) and an interaction between group and modality (*F*_1_,_572_ = 31.9, *p* > 0.0001). The group effect was significant in the auditory-only condition (*B* = 10.74, *t*_35_ = 3.781, *p* = 0.0005), but not in the audiovisual condition (*B* = 2.58, *t*_35_ = 0.909, *p* = 0.3696). No other variables interacted with group. The poorer performance of participants tested in their homes may reflect a limited control of the test environment. It may also reflect the difference in age of the participants, as the adults tested at home had a mean age of 43 years (SD = 4.3 years), and the adults tested in the lab had a mean age of 22.3 years (SD = 2.4 years). Additionally, we tested hearing detection thresholds in the lab but not at home, so it is possible that the difference is due to inaccurate self-reports of normal hearing among the adults tested at home.

The lower overall performance of the ANH tested at −10 dB SNR allows for a performance-matched comparison to the CHL. A mixed-effects linear model was used with fixed effects and interactions of group, mask, and modality, and a random intercept per subject. From this model, we examined the effects of group and interactions between group and each within-subjects variable. Model estimates and *post hoc* comparisons are shown in Supplementary Table 3. There was a marginal overall group effect (*F*_1,26_ = 3.5, *p* = 0.0723), with the ANH at −10 dB SNR performing slightly worse overall than the CHL at 0 dB SNR. The group effect was not significant in the auditory-only baseline condition (*B* = −6.23, *t*_41_ = −1.38, *p* = 0.1724), but there were significant interactions of modality and mask condition (*F*_4,471_ = 39.2, *p* < 0.0001), modality and group (*F*_1,471_ = 13.4, *p* = 0.0003), and mask and group (*F*_4,471_ = 8.1, *p* < 0.0001). The interaction of modality and group was driven by greater audiovisual benefit in the ANH than in the CHL (*B* = 6.62, *t*_471_ = 3.66, *p* = 0.0003). The interaction of group and mask condition was driven by the fact that the fabric mask degraded performance more in the ANH than in the CHL (*B* = 13.91, *t*_471_ = 4.833, *p* < 0.0001). The interaction of fabric mask and group is consistent with our hypothesis that the CHL may be less impacted by the acoustic attenuation of the face mask, because their hearing loss reduces access to high-frequency cues even in the no-mask condition. However, there is no relationship between individual differences in relative degradation caused by the fabric mask and either CHL’s aided SII or 4 kHz thresholds. This could be due to the delay between audiometric testing and the study tasks. It is also possible that this relationship may emerge with a larger sample of CHL.

## Auditory-Only and Audiovisual Phonetic Feature Transmission

**FIGURE 6:**
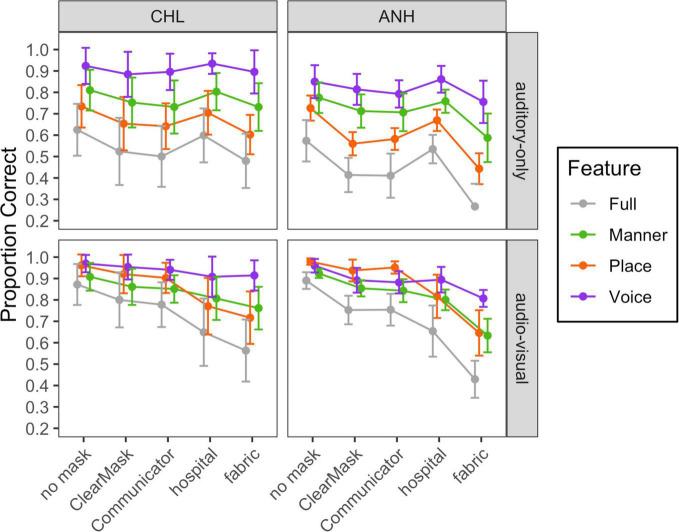
Mean and standard deviation of scores for each of the auditory-only and audiovisual stimulus conditions, plotted separately for each group and modality, as indicated at the top and right of each panel, respectively. Mask type is indicated on the horizontal axis, and articulatory feature is indicated with color, as defined in the legend.

We conducted additional analyses to determine which speech features were impacted by the face masks. Using data from the CHL and ANH tested at −10 dB SNR, we examined the effects of face masks on the perception of three speech features: voicing, place of articulation, and manner of articulation. Table 1 shows feature classification for each consonant. Other data sets were excluded from this analysis because of the common occurrence of ceiling performance. Figure 6 demonstrates the mean and distribution of voicing, place, and manner feature transmission accuracy in each mask condition in auditory-only (top) and audiovisual (bottom) conditions for the CHL (left) and the ANH (right). The full consonant accuracy data are provided in gray for reference.

**TABLE 1:** Phonetic feature assignment.

Voicing		
Voiced	Unvoiced	
b, d, m, n, v, z	s, h, k, t, p, ∫	

**Manner**		
Stop	Fricative	Nasal
b, d, p, t, k	v, z, s, h, ∫	m, n

**Place**		
Front	Middle	Back
p, b, m, v	s, z, t, d, n	∫, k, h

For each modality and group, a linear mixed-effects model with a random intercept per subject was used to analyze the effects and interactions of feature and mask conditions on RAU-transformed feature transmission data. The no mask condition and place feature served as the reference condition. Reference conditions were systematically varied as needed for *post hoc* comparisons.

In both groups, the auditory-only transmission was best for the voicing feature and worst for the place feature. Rank differences between the face mask conditions for each phonetic feature were similar to the differences in consonant recognition accuracy. These findings were confirmed by two statistical models. The final model of the feature transmission data in the CHL included main effects of mask (*F*_4_,_453_ = 20.6, *p* < 0.0001) and feature (*F*_2_,_453_ = 423.6, *p* < 0.0001) and no significant interaction. Accuracy was lower for place than manner (*t*_453_ = 9.989, *p* < 0.0001) and lower for manner than voicing (*t*_453_ = 18.682, *p* < 0.001).

The final model for the ANH tested at −10 dB SNR in auditory-only conditions included main effects of mask (*F*_4_,_279_ = 47.5, *p* < 0.0001) and feature (*F*_2_,_279_ = 190.1, *p* < 0.0001), and an interaction of mask and feature (*F*_8_,_279_ = 2.5, *p* = 0.0122). Model estimates and *post hoc* comparisons are shown in Supplementary Table 4. With no mask, accuracy was lower for place than manner (*t*_453_ = 9.989, *p* < 0.0001) and lower for manner than voicing (*t*_453_ = 18.682, *p* < 0.001). The interaction of mask condition and feature reflects the fact that face masks impacted perception of place more than manner and voicing. More specifically, the hospital mask only impacted perception of the place feature. The other masks impacted perception of all features, but the ClearMask™ and fabric masks impacted place perception more than voicing (*B* = 10.27, *t*_279_ = 2.648, *p* = 0.0086; *B* = 14.82, *t*_279_ = 3.822, *p* = 0.0002) and manner (*B* = 9.94, *t*_279_ = 2.563, *p* = 0.0109; *B* = 8.33, *t*_279_ = 2.149, *p* = 0.0325). The impact of the Communicator™ was also marginally greater for place than voicing (*B* = 7.06, *t*_279_ = 1.842, *p* = 0.0666). Finally, the impact of the fabric mask was marginally greater for manner than voicing (*B* = 6.49, *t*_279_ = 1.673, *p* = 0.0954).

Audiovisual feature transmission data from the CHL tested at 0 dB SNR and the ANH tested at −10 dB SNR are shown in the bottom half of Figure 6. Rank differences between the face mask conditions for each phonetic feature were similar to the differences in consonant recognition accuracy. However, there was a notable difference in the patterns of feature transmission between conditions in which the participants could see the talker’s mouth region (no mask, ClearMask™, and Communicator™ conditions) and conditions in which the mouth region was obscured (fabric and hospital mask conditions). These findings were confirmed with two statistical models.

The final audiovisual model for the CHL included effects of mask (*F*_4,427_ = 72.8, *p* < 0.0001) and feature (*F*_2,427_ = 101.2, *p* < 0.0001), and an interaction of mask condition and feature (*F*_8,427_ = 8.1, *p* < 0.0001). Model estimates and *post hoc* comparisons are shown in Supplementary Table 5. The interaction reflects the fact that the difference in transmission between conditions in which the participants can see the talker’s mouth region and conditions in which the mouth region is obscured was larger for place than voicing and manner (*B* ≥ 13.65, *t*_427_ ≥ 3.389, *p* ≤ 0.0007).

The final audiovisual model for the ANH tested at −10 dB SNR included the effects of mask (*F*_4,276_ = 135.3, *p* < 0.0001) and feature (*F*_2,276_ = 50.2, *p* < 0.0001). The effect of feature varied significantly across mask conditions (*F*_8,276_ = 10.7, *p* < 0.0001). Model estimates and *post hoc* comparisons are shown in Supplementary Table 6. As in the CHL, the difference in transmission between conditions in which the participants could see the talker’s mouth region and conditions in which the mouth region was obscured was larger for place than voicing and manner (*B* ≥ 12.76, *t*_276_ ≥ 3.135, *p* ≤ 0.0019). The difference between the fabric mask and the two transparent masks was also greater for manner than voicing (*B* ≥ 11.65, *t*_276_ ≥ 2.861, *p* ≤ 0.0045).

## Visual-Only Phonetic Feature Transmission

**FIGURE 7:**
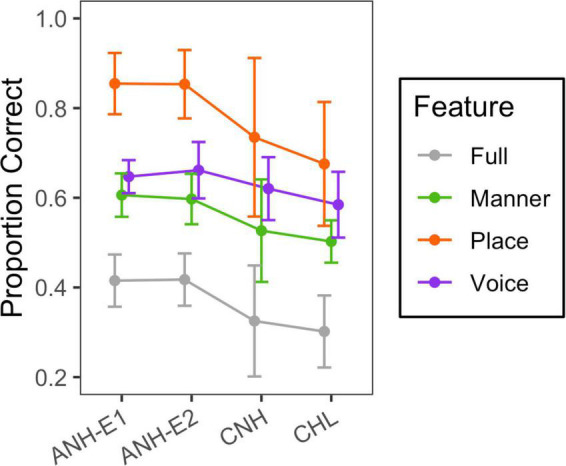
Mean and standard deviation of scores for the visual-only condition. Group is indicated on the horizontal axis, and phonetic feature is indicated with color, as defined in the legend.

Visual-only data were obtained for 11/16 CNH, 12/18 CHL, and 16/23 ANH in Experiment 1, as well as 15/15 ANH in Experiment 2. Visual-only consonant recognition accuracy data are plotted in gray in Figure 7. Mean accuracy was 31% for the group of CNH, 33% for the group of CHL, and 42% for both ANH groups. Welch’s *t*-tests indicated greater visual-only consonant recognition accuracy in the ANH than in the CNH (*t*_24_ = 4.695, *p* < 0.0001) and the CHL (*t*_26_ = 3.257, *p* = 0.0031) but no difference between the CNH and the CHL (*t*_41_ = 0.286, *p* = 0.7761).

Visual-only feature transmission data are plotted in color in Figure 7. This figure shows higher transmission of the place feature than the voicing and manner features and higher transmission in the ANH than in the CNH and CHL. Additionally, there was large variability in place transmission among children.

A linear mixed-effects model with a random intercept per subject was used to analyze the effects and interactions of feature and group on RAU-transformed visual-only feature transmission data. We combined the adults tested at home and the adults tested in the lab into one group. The CHL group and place feature served as the reference condition. Reference conditions were systematically varied as needed for *post hoc* comparisons. Model estimates and *post hoc* comparisons are shown in Supplementary Table 7. There were significant effects of group (*F*_2,51_ = 12.4, *p* < 0.0001) and feature (*F*_2,269_ = 175, *p* < 0.0001), as well as an interaction of group and feature (*F*_4,269_ = 6.7, *p* < 0.0001). In all the groups, visual-only feature transmission was higher for place than for voicing (*B* ≤ −11.42, *t*_269_ ≥ −4.321, *p* < 0.0001) and manner (*B* ≤ −19.18, *t*_269_ ≤ −7.254, *p* < 0.0001), and higher for voicing than for manner (*B* ≥ 4.98, *t*_269_ ≥ 2.933, *p* ≤ 0.0036). However, it is important to note the lack of independence between features (for example, all nasal sounds are voiced), and that the chance of correct transmission of voicing is greater than the chance of correct transmission of place and manner.

Feature transmission accuracy was higher in the ANH than in the CNH for place (*B* = 19.54, *t*_103_ = 6.155, *p* < 0.0001), manner (*B* = 9.78, *t*_103_ = 3.081, *p* = 0.0026), and voicing (*B* = 7.01, *t*_103_ = 2.207, *p* = 0.0295). Feature transmission accuracy was higher in the adults than in the CHL for place (*B* = 13.63, *t*_97_ = 4.519, *p* < 0.0001) and manner (*B* = 7.148, *t*_97_ = 2.369, *p* = 0.0198). The difference between age groups was greater for place than for voicing (*B* ≥ 10.45, *t*_269_ ≥ 3.707, *p* ≤ 0.0003) and manner (*B* ≥ 6.49, *t*_269_ ≥ 2.302, *p* ≤ 0.0221). There were no differences in visual-only feature transmission between the CNH and the CHL.

## Discussion

The purpose of this study was to determine the impact of four types of face masks on auditory and audiovisual speech recognition in noise among CHL, CNH, and ANH. We compared performance with a hospital mask, a fabric mask, a primarily transparent mask (ClearMask™), and a primarily opaque disposable mask with a transparent window (The Communicator™) to baseline conditions with no mask. We expected the face masks to reduce transmission of high-frequency speech content and, thus, impact discrimination of consonants in noise, especially consonant features that rely on high-frequency acoustic cues, such as place of articulation. We hypothesized that the impact of high-frequency acoustic attenuation depends on audibility, such that there is little impact on the masks in CHL with poor high-frequency audibility. In audiovisual conditions, we also hypothesized that the loss of visual cues associated with the opaque masks would impact discrimination of consonants in noise, especially consonant features that are easier to speech-read, such as place of articulation. Thus, we expected the children with poor high-frequency audibility to do best with the transparent masks. However, given that previous studies have shown greater acoustic attenuation in transparent masks than opaque (hospital and fabric) masks (Atcherson et al., 2020; Corey et al., 2020; Vos et al., 2021), it was uncertain how the participants with good high-frequency audibility would fare in the trade-off between loss of visual cues and acoustic attenuation.

### Effects of Face Masks on Speech Acoustics

We measured the acoustic power spectra of speech produced by the talker at baseline and while wearing each mask. These acoustic measurements demonstrated that the masks caused high-frequency attenuation. As in previous studies, the hospital mask caused the least attenuation (e.g., Atcherson et al., 2020; Bottalico et al., 2020; Corey et al., 2020; Goldin et al., 2020; Vos et al., 2021). Although previous studies have shown greater high-frequency acoustic attenuation from transparent masks than from fabric masks (Corey et al., 2020; Brown et al., 2021), the fabric mask used in our study caused the greatest attenuation. The acoustic transmission properties of fabric masks vary considerably depending on type of fabric and number of fabric layers. One study showed 0.4- to 9-dB greater attenuation between 2 and 16 kHz for fabric masks than for a disposable hospital mask (Corey et al., 2020). The double-layered mask used in this study resulted in 7.4-dB greater attenuation between 2 and 16 kHz than the hospital mask, suggesting that our fabric mask is on the higher end of acoustic attenuation range.

### Effects of Face Masks on Adults’ Speech Perception in Noise

In the perception experiments, we found that face masks degraded consonant recognition in noise. The relative impact of each mask was consistent across the groups of adults tested at home and in the lab, as well as across SNRs. The impact of the masks on auditory-only consonant recognition in noise varied in accordance with the high-frequency acoustic attenuation caused by the masks. The hospital mask had the least high-frequency acoustic attenuation and least impact on performance; the fabric mask had the greatest high-frequency acoustic attenuation and greatest impact on performance. The differences in the relative impact of the masks are consistent with other studies comparing between effects of fabric and hospital masks (Bottalico et al., 2020) or between effects of a hospital mask and the ClearMask™ (Yi et al., 2021) on auditory-only perception in noise or babble.

In audiovisual conditions, the two transparent masks (ClearMask™ and Communicator™) had the least impact on performance, even though acoustic attenuation is greater for the transparent masks than for the hospital mask. Thus, in the tradeoff between loss of visual cues and high-frequency acoustic attenuation, visual cues seem to be more important for consonant recognition. One caveat is that the test configuration, a close, frontal view of the talker’s face, short test sessions, and CV stimuli, may not be representative of listening in daily life. These results are consistent with a previous study that compared the effects of a hospital mask and the ClearMask™ on the perception of audiovisual speech in noise and babble (Yi et al., 2021). In that study, the results depended on the masker and speaking style. The ClearMask™ impacted the perception of clear speech in babble, conversational speech in noise, and conversational speech in babble but not the perception of clear speech in noise. The hospital mask impacted perception more than the ClearMask™ when speech was spoken in a clear style in babble, in a clear style in noise, or in a conversational style in noise but not in a conversational style in babble.

The finding that visual cues available with a transparent mask outweigh the additional acoustic attenuation diverges from published results (Brown et al., 2021). Brown et al. (2021) compared audiovisual sentence recognition accuracy among a hospital mask, a cloth mask with a paper filter, a cloth mask without a paper filter, and a cloth mask with a transparent window. In moderate (−5 dB SNR) and high (−9 dB SNR) levels of noise, the hospital mask impacted performance the least, followed by the cloth mask with no paper filter. The cloth mask with a paper filter and the cloth mask with a transparent window had the greatest impact. The cloth mask with a filter seemed to have caused slightly less attenuation than the transparent mask. Therefore, the fact that the transparent mask and the cloth mask with a filter similarly impacted performance may represent a small audiovisual benefit from the transparent window. However, unlike in our study, the benefit of the transparent window was not large enough to overcome the detrimental effects of high-frequency attenuation relative to the hospital mask. In other words, the availability of visual cues from the transparent window did not outweigh the added acoustic attenuation.

There are several differences between the Brown et al. (2021) study and this study that could account for the difference in results. First, the type of transparent mask differed between the two studies. Second, we simulated the effects of masks on speech acoustics and visual speech cues, whereas Brown et al. (2021) audio-visually recorded speech stimuli in each mask. Therefore, the participants in our study had consistent access to a full view of the mouth, whereas those in the previous study (and in natural conversations) may have been affected by improper mask placement and/or fogging. Furthermore, our stimuli did not capture phoneme-specific effects of the masks on articulation, including any targeted adjustments to articulation that talkers might make in response to wearing a mask (Cohn et al., 2021). This is important, because 60% of survey respondents report communicating differently when wearing a mask, including changing their manner of speaking, minimizing linguistic content, and using gestures, facial expressions, and eye contact more often and more purposefully than when communicating without a face mask (Saunders et al., 2020). Finally, we examined perception of consonants in CV syllables, whereas Brown et al. (2021) examined perception of words in sentences. Unlike this study, the stimuli used by Brown et al. (2021) include linguistic context and may include additional visual cues to prosody, such as rigid head movements (Munhall et al., 2004; Davis and Kim, 2006).

### Effect of Face Masks on Children’s Speech Perception in Noise

The effects of face masks on speech perception in noise for the CNH did not differ from those for the ANH. High-frequency acoustic attenuation impacted children’s auditory speech sound recognition, consistent with previous studies on children’s susceptibility to the detrimental effects of decreased bandwidth (Stelmachowicz et al., 2000, 2001; Mlot et al., 2010; McCreery and Stelmachowicz, 2011) and with previous data on the impact of face masks on auditory-only speech recognition in CNH (Flaherty, 2021; Sfakianaki et al., 2021). Although we observed no effects of age group, it is possible that these effects would emerge in younger children. Our exploration of the impact of child age suggested that younger children may be more impacted by face masks in auditory-only conditions. However, it was impossible to separate age effects from ceiling effects in the current child data. Additional data are needed to determine how the negative impact of face masks varies as a function of age.

We expected that CHL with poor high-frequency audibility would be less impacted by high-frequency acoustic attenuation caused by the masks than children with good high-frequency audibility. However, we did not observe a difference in the impact of high-frequency acoustic attenuation between the CHL and the CNH. Overall, the CHL in this study had relatively good aided audibility, with a mean aided SII score of 80.8 at 65 dB SPL. It is possible that a more heterogeneous sample of CHL, including those with poorer high-frequency aided audibility, would have a smaller detrimental effect of face masks in the auditory-only condition.

### Audiovisual Benefit With Face Masks

When the mouth region was visible, there was significant audiovisual benefit in all the groups, with the greatest benefit in the ANH and the least benefit in the CNH. The finding that CNH benefited less than ANH is consistent with previous research (Wightman et al., 2006; Lalonde and Holt, 2016; Lalonde and McCreery, 2020). However, the finding that CHL benefited less than ANH conflicts with our previous study, which showed similar benefits in ANH and CHL (Lalonde and McCreery, 2020).

In this study, we found that audiovisual benefit was greatest when the mouth was visible, consistent with research on the importance of the mouth region for lip-reading. The difference in benefit between the opaque and transparent masks is consistent with previous findings from adults indicating that there is a minimal difference between lip-reading accuracy when viewing a whole face and viewing only the lips or the lower half of the face (Greenberg and Bode, 1968; Ijsseldijk, 1992; Marassa and Lansing, 1995).

Most of the groups in this study also showed a small yet significant audiovisual benefit with the opaque face masks. This benefit was observed with the hospital and fabric masks in the CHL and ANH tested at home, and the ANH tested in the lab at −10 dB SNR, as well as with the fabric mask in the ANH tested in the lab at 0 dB SNR. These findings are consistent with those of Yi et al. (2021) who observed a significant audiovisual benefit with the ClearMask™ in ANH tested in noise and a significant audiovisual benefit with both the ClearMask™ and a hospital mask in ANH tested in babble. The finding of audiovisual benefit with opaque masks is consistent with studies suggesting that non-oral regions of the face contain subtle visual cues that contribute to audiovisual speech recognition in noise (Summerfield, 1979; Scheinberg, 1980; Benoît et al., 1996; Preminger et al., 1998; Thomas and Jordan, 2004; Davis and Kim, 2006; Jordan and Thomas, 2011). For example, although occluding the bottom half of the face decreases visual-only recognition and audiovisual enhancement of CVs, visual-only recognition accuracy remains above chance, and a small audiovisual enhancement remains for some CVs (Jordan and Thomas, 2011). Thomas and Jordan (2004) suggested that occluding the lower half of the face might affect viewing and attention strategies. Non-oral face and head motions become more important when oral cues are not available; therefore, these cues could play a substantial role in understanding audiovisual speech with face masks (Thomas and Jordan, 2004; Jordan and Thomas, 2011). The significant audiovisual benefit we observed, despite the presence of an opaque face mask, suggests that talkers should try to keep their face visible to listeners in noisy conditions, even when wearing an opaque mask, as many listeners will benefit from limited visual cues available.

### Effects of Face Masks on Phonetic Feature Transmission

We measured the effects of face masks on phonetic feature transmission to determine the type of perceptual errors face masks are likely to cause. Rank differences between performance with each mask were the same for all three consonant features as for overall consonant recognition accuracy. However, the face masks impacted the perception of some consonant features more than others.

We expected consonant features based primarily on high-frequency acoustic cues, such as the place of articulation of voiceless fricatives and stops (Stevens, 2000), to be most impacted by face masks. In the adults tested at −10 dB SNR, this expectation was confirmed. The masks affected adults’ perception of auditory-only place more than voicing and manner. In CHL, the effect of the masks on auditory perception did not differ across features. However, more complex models are required to confirm the significance of this difference in patterns. In audiovisual conditions, we also expected consonant features that are easier to lip-read, such as place of articulation (Binnie et al., 1974; Owens and Blazek, 1985), to be most impacted by the opaque mask. This expectation was confirmed in both the CHL and the ANH; there was a greater difference between the opaque masks and transparent masks for place than for voicing and manner.

Although baseline performance in the auditory-only condition did not differ between the CHL tested at 0 dB SNR and the ANH tested at −10 dB SNR, the feature transmission patterns differed. The CHL tested at 0 dB SNR showed greater differences between the perception of voicing and other features than the ANH tested at −10 dB SNR. In other words, the increased masking noise used to decrease ANH performance to the level of CHL likely resulted in an error pattern different than that resulting from the combination of noise and children’s hearing loss. Given that voicing is poorly transmitted visually, the visual signal may have less potential benefit to consonant perception in the ANH tested at –10 dB SNR than the CHL tested at 0 dB SNR. In other words, differences in audiovisual benefit across groups who are tested at different SNRs may reflect differences in patterns of acoustic errors, in addition to differences in the ability to use visual speech information.

### Visual-Only Consonant Perception in Children With Hearing Loss, Children With Normal Hearing, and Adults With Normal Hearing

Our results demonstrated that ANH are better at visual-only consonant recognition than CNH and CHL. There was large variability in visual-only consonant perception in the children, especially in the transmission of the place feature, but there was no difference in visual-only consonant perception between the children with and without hearing loss. Previous studies have provided conflicting results regarding whether CHL are better at lip-reading than CNH. Although some studies have shown no effect of hearing status on lip-reading (Conrad, 1977; Kyle et al., 2013), others have shown that a subset of CHL has better lip-reading than CNH (Lyxell and Holmberg, 2000; Kyle and Harris, 2006; Tye-Murray et al., 2014). Additional research is needed to understand the factors underlying these conflicting findings.

## Limitations and Future Directions

This study examined the perceptual consequences of the acoustic attenuation and visual obstruction caused by a variety of face masks. However, the test configuration in this study, a close frontal view of the talker’s face, short test sessions, CV stimuli, and simulated masks, may not be representative of listening in daily life. The mask simulations do not capture any phoneme-specific effects of masks on articulation, including targeted adjustments to articulation that talkers might adopt when wearing a face mask. The mask simulations also do not account for the effects of improper mask placement and/or fogging. In future studies, it would be helpful to compare the acoustic consequences and perception of speech produced while wearing a mask to speech with mask simulations. Future studies could also test the perception of consonants, words, and sentences using the same participants and target talker to see how well results for consonant perception might generalize to the type of speech we encounter in everyday life.

We tested children from a broad age range, but ceiling effects precluded an examination of developmental differences in the negative impact of face masks. In future studies, a larger sample of children and performance-matching techniques would allow us to examine how the negative impact of face masks varies over development, and a more heterogeneous sample of CHL would allow us to probe whether the impact of acoustic attenuation caused by face masks depends on high-frequency aided audibility. A better understanding of children’s orienting behaviors in classrooms would allow us to make more concrete recommendations. The combination of noise and face masks negatively impacts speech understanding in children. Future studies could examine the impact of masks on children in other listening conditions, such as conversational (rather than clear) speech (Brown et al., 2021; Smiljanic et al., 2021; Yi et al., 2021), listening to speech produced by a non-native talker (Smiljanic et al., 2021), or listening to speech in one’s non-native language. Future studies could include children who use cochlear implants, as their deficits in acoustic-phonetic access differ qualitatively from children who use hearing aids.

## Conclusion

• The best face masks for promoting speech understanding depend on whether visual cues will be available. When listeners will not be able to view the talker, a hospital mask is the best option for ANH, CNH, and CHL. Fabric masks that cause less acoustic attenuation than the one used in the current study (those made with fewer layers and less dense fabric) could be another good option. From a communication perspective, when working one-on-one and face-to-face with a listener at a short distance, a well-fit ClearMask™ is the best option, as it provides visual speech cues that more than compensate for higher levels of acoustic attenuation.
• In a classroom setting, the best mask for promoting speech understanding will depend on the degree to which children orient to the talker and whether the teacher is positioned to provide visual cues for all students. Previous studies have shown that young CNH do not consistently orient to the target talker the way adults do (Ricketts and Galster, 2008; Valente et al., 2012; Lewis et al., 2015, Lewis et al., 2018). The hospital mask may be better when communicating with children who do not orient to the talker.
• Even when a talker wears an opaque mask, many listeners benefit from seeing the head and facial movements that are not obscured by the mask. Therefore, talkers wearing opaque masks should try to keep their faces visible.
• One caveat to these conclusions is that the test configuration in this study, a close frontal view of the talker’s face, short test sessions, CV stimuli, and simulated masks, may not be representative of listening in daily life.

## Data Availability Statement

The datasets and stimuli presented in this study can be found at https://osf.io/5wapg/.

## Ethics Statement

The studies involving human participants were reviewed and approved by the Institutional Review Board at Boys Town National Research Hospital. Written informed consent to participate in this study was provided by the participants or their legal guardians. Written informed consent was obtained from the individual(s) for the publication of any identifiable images or data included in this article.

## Author Contributions

All authors contributed to conception and design of the study. LL secured the funding and assembled the research team. KL and EB created the stimuli and performed the data analyses. MM recruited and collected the data for Experiment 1. KL led the recruitment and data collection for Experiment 2. KL, EB, and MM wrote sections of the manuscript. All authors contributed to the manuscript revision, and read and approved the submitted version.

## Conflict of Interest

The authors declare that the research was conducted in the absence of any commercial or financial relationships that could be construed as a potential conflict of interest.

## Publisher’s Note

All claims expressed in this article are solely those of the authors and do not necessarily represent those of their affiliated organizations, or those of the publisher, the editors and the reviewers. Any product that may be evaluated in this article, or claim that may be made by its manufacturer, is not guaranteed or endorsed by the publisher.

## Abbreviations

CHL: children with hearing loss
CNH: children with normal hearing
ANH: adults with normal hearing
SNR: signal-to-noise ratio
CDC: Centers for Disease Control and Prevention.
