# Supplementary material for: Face Masks Impact Auditory and Audiovisual Consonant Recognition in Children With and Without Hearing Loss
Source: Front Psychol. 2022 May 13;13:874345. doi: 10.3389/fpsyg.2022.874345 (PMC9137424; doi:10.3389/fpsyg.2022.874345)
Supplement: Supplementary file 1 [file Table_1.docx]

**Supplementary Materials**

**Visual Mask Simulation**

***Extracting Face Motion Data.*** Video files were processed using custom software that automatically marks the position of 66 points on the face in each frame of the video (Supplemental Figure 1; Saragih, Lucey & Cohn, 2011). Markers include 17 points at the perimeter of the lower half of the face.  The unedited FaceScanner data are saved in a structure called “Original_FSdata_ChooseCV.m” available at https://osf.io/5wapg/.

***Simulating Masks.*** Simulated masks were created by inserting a filled white polygon onto each video image. The simulated opaque masks (hospital, cloth) were created based on the location of the 17 markers on the perimeter of the lower half of the face (markers 0 to 16 in) and a marker on the bridge of the nose (marker 28) (see Supplemental Figure 1A). To completely cover the jaw line, the mask extended 25 pixels below the y-axis location of the points on the perimeter of the face (video dimensions: 1920 x 1080 pixels; mean face width = 472 pixels).


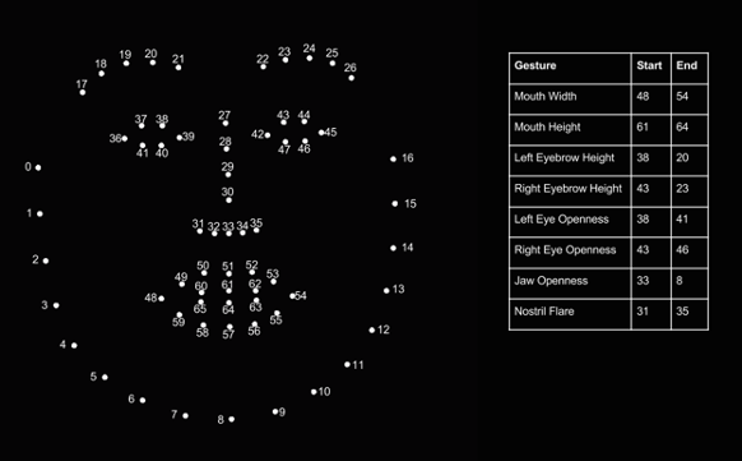

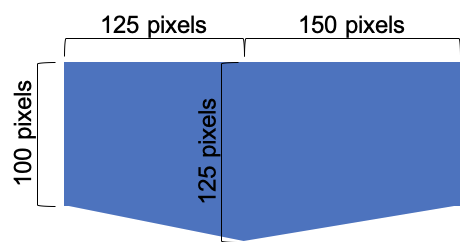
The simulated Communicator^TM^ was created in a similar fashion, except that two filled polygons were used to create the effect of a cutout in the middle of the simulated fabric mask shape. The cutout was a pentagon shape, with 300 pixels width, 100 pixels height at the edges, and 125 pixels height at the center. The center of the cutout was located at the x-coordinate of the nose marker (28) and at the y-coordinate halfway between the y-coordinates of markers at the tip of nose (marker 33) and the center of upper lip (marker 51). Because the cutout was subjectively off-center relative to the talker’s mouth, we created a second asymmetrical cutout (Supplemental Figure 1B). The cutout was an asymmetrical pentagon shape, with 275 pixels width, 100 pixels height at the edges, and 125 pixels height at the maximum. The center of the cutout was located at the x-coordinate of the marker at the upper middle of the bridge of the nose and at the y-coordinate halfway between the markers at the central tip of the nose and the center of the upper lip. Three observers indicated preference for the asymmetric cutout over the symmetric one.

(B)

(A)

**Supplemental Figure 1.** (A) Diagram of the 66 points on the face automatically tracked using specialized software. (B). Dimensions of the cutout used to simulate the Communicator^TM^.

***Adjustments to Face Masks.*** An independent observer was asked to note any problems with the simulated face masks. She noted that the simulated face mask moved when the talker blinked and that there was frame-to-frame jitter in the simulated facemask.

***Correcting For Blinks.*** To correct for effects related to blinking, we plotted frame-by-frame variation in left eye openness (as calculated automatically in the software based on markers on the left eyebrow and the upper eyelid) (Supplemental Figure 2A, blue). Next, we calculated frame-to-frame change in left eye openness (Supplemental Figure 2A, red) and located minima in change in left eye openness (points with maximum velocity of decreasing eye openness) using outlier analysis and visual inspection of the plots.

Supplemental Figure 2B shows a video with 2 blinks, which can be seen from the decrease in eye openness (blue) near frames 11 and 50. The scaled y-coordinate of the nose marker (marker 28) is shown in green. There are transient increases in the y-coordinate of the nose marker during these blinks. To remove these transient increases, we replaced the y-coordinate of the nose marker 28 during each blink with a linear estimation based on the y-coordinates of the same marker from frames immediately preceding and following the blink (as shown in purple). Y-coordinate data from 11 frames per blink were replaced, beginning 3 frames before the local maximum velocity of decreasing eye openness. The topmost points from the left and right side of the perimeter of the face were edited in the same manner.

After one independent observer noted that the blink-related mask movement was still visible in 6 of 36 videos, the x-coordinate data were edited in the same manner as the y-coordinate data. After these corrections, one observer noted that there was still a problem with one video. We discovered that in video di_3, the bottom of the mask seemed to move with the final blink. We applied the blink corrections to all of the remaining markers in the mask for this video.

***Correcting for Marker Position Jitter.*** To deal with the jitter, we replaced data from both coordinates with a 3-frame moving window average of the same data. (Frames 1 is the average of the first 3 frames, the final frame is the average of the last 3 frames, and all frames in between are the average of the current frame and the one preceding and following it.) Supplemental Figure B shows one example of this correction. The jittery original data are shown in blue and red, and the edited data are shown in green and purple.

Of the three independent observers, one indicated that two videos still included jitter. To fix the remaining jitter in those three videos, we plotted the x- and y-coordinate locations a function of frame number for all markers use to create the mask, visually located the portions of the videos that still included jitter, and averaged over a greater number of frames for that portion of the video. Specifically, in video di_1, we averaged the first 10 frames over 7 frames. In shi_1, we averaged over 5 frames for the full duration of the video.

The code used to create the simulated face masks is available online, along with the simulated face mask stimuli (https://osf.io/5wapg/).

Supplemental Figure 2. (A). Example adjustments to mask marker positions correcting for blinks. (B). Example adjustments to mask marker positions correcting for position jitter.

(B)

(A)


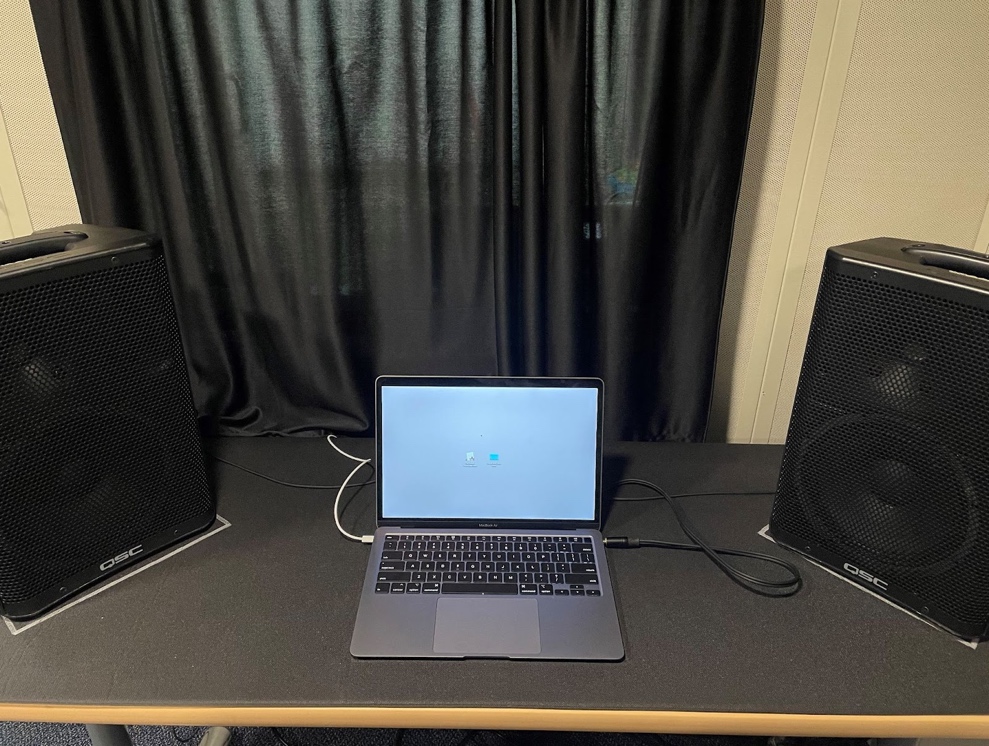


Supplemental Figure 3. Hardware used for remote testing.

| Supplemental Table 1. Linear model estimates and post hoc comparisons for consonant recognition accuracy in Experiment 1. Reference conditions are on the left. | | | | | |
| --- | --- | --- | --- | --- | --- |
|  | **Estimate** | **S. E.** | **df** | **t-value** | **p-value** |
| **Group x Modality Interaction** |  |  |  |  |  |
| CNH - CHL: AO - AV | 5.95 | 1.535 | 916 | 3.874 | 0.0001 |
| CNH - ANH: AO - AV | 8.87 | 1.447 | 916 | 6.131 | < 0.0001 |
| ANH - CHL: AO - AV | -2.92 | 1.367 | 916 | -2.140 | 0.0326 |
| **Effect of Group (per Modality)** |  |  |  |  |  |
| ***Auditory-Only*** |  |  |  |  |  |
| CNH - CHL | -23.06 | 3.223 | 62 | -7.155 | < 0.0001 |
| CNH - ANH | -0.85 | 3.051 | 61 | -0.280 | 0.7805 |
| ANH - CHL | -22.20 | 2.940 | 61 | -7.552 | < 0.0001 |
| ***Audio-Visual*** |  |  |  |  |  |
| CNH - CHL | -17.11 | 3.227 | 62 | -5.303 | < 0.0001 |
| CNH - ANH | 8.02 | 3.056 | 62 | 2.623 | 0.0110 |
| ANH - CHL | -25.13 | 2.946 | 61 | -8.528 | < 0.0001 |
| **Mask x Modality Interaction** |  |  |  |  |  |
| No Mask - ClearMask^TM^ | 0.92 | 1.858 | 916 | 0.493 | 0.6219 |
| No Mask - Communicator^TM^ | 1.49 | 1.851 | 916 | 0.805 | 0.4213 |
| No Mask - Hospital | -19.50 | 1.853 | 916 | -10.523 | < 0.0001 |
| No Mask - Fabric | -19.03 | 1.858 | 916 | -10.244 | < 0.0001 |
| ClearMask^TM^ - Communicator^TM^ | 0.57 | 1.827 | 916 | 0.314 | 0.7539 |
| ClearMask^TM^ - Hospital | -20.42 | 1.829 | 916 | -11.163 | < 0.0001 |
| ClearMask^TM^ - Fabric | -19.95 | 1.833 | 916 | -10.882 | < 0.0001 |
| Communicator^TM^ - Hospital | -20.99 | 1.823 | 916 | -11.516 | < 0.0001 |
| Communicator^TM^ - Fabric | -20.52 | 1.827 | 916 | -11.233 | < 0.0001 |
| Hospital - Fabric | 0.47 | 1.829 | 916 | 0.255 | 0.7990 |
| **Effect of Mask (per Modality)** |  |  |  |  |  |
| ***Auditory-Only*** |  |  |  |  |  |
| NoMask - ClearMask^TM^ | -8.15 | 1.286 | 916 | -6.337 | < 0.0001 |
| NoMask - Communicator^TM^ | -10.99 | 1.277 | 916 | -8.605 | < 0.0001 |
| No Mask - Hospital | -2.17 | 1.279 | 916 | -1.700 | 0.0895 |
| NoMask - Fabric | -14.51 | 1.283 | 916 | -11.312 | < 0.0001 |
| ClearMask^TM^ - Communicator^TM^ | -2.84 | 1.291 | 916 | -2.200 | 0.0280 |
| ClearMask^TM^ - Hospital | 5.98 | 1.293 | 916 | 4.620 | < 0.0001 |
| ClearMask^TM^ - Fabric | -6.36 | 1.296 | 916 | -4.907 | < 0.0001 |
| Communicator^TM^ - Hospital | 8.81 | 1.284 | 916 | 6.863 | < 0.0001 |
| Communicator^TM^ - Fabric | -3.52 | 1.287 | 916 | -2.736 | 0.0063 |
| Hospital - Fabric | -12.34 | 1.290 | 916 | -9.562 | < 0.0001 |
| ***Audio-Visual*** |  |  |  |  |  |
| NoMask - ClearMask^TM^ | -7.23 | 1.342 | 916 | -5.391 | < 0.0001 |
| NoMask - Communicator^TM^ | -9.50 | 1.342 | 916 | -7.079 | < 0.0001 |
| No Mask - Hospital | -21.67 | 1.342 | 916 | -16.156 | < 0.0001 |
| NoMask - Fabric | -33.54 | 1.345 | 916 | -24.946 | < 0.0001 |
| ClearMask^TM^ - Communicator^TM^ | -2.27 | 1.293 | 916 | -1.753 | 0.0800 |
| ClearMask^TM^ - Hospital | -14.44 | 1.293 | 916 | -11.167 | < 0.0001 |
| ClearMask^TM^ - Fabric | -26.31 | 1.296 | 916 | -20.297 | < 0.0001 |
| Communicator^TM^ - Hospital | -12.18 | 1.293 | 916 | -9.414 | < 0.0001 |
| Communicator^TM^ - Fabric | -24.05 | 1.297 | 916 | -18.547 | < 0.0001 |
| Hospital - Fabric | -11.87 | 1.296 | 916 | -9.157 | < 0.0001 |
| **Effect of Modality (per Mask and Group)** | |  |  |  |  |
| ***NoMask*** |  |  |  |  |  |
| CNH | 20.86 | 1.657 | 916 | 12.590 | < 0.0001 |
| CHL | 26.81 | 1.585 | 916 | 16.920 | < 0.0001 |
| ANH | 29.73 | 1.488 | 917 | 19.982 | < 0.0001 |
| **ClearMask^TM^** |  |  |  |  |  |
| CNH | 21.78 | 1.621 | 916 | 13.437 | < 0.0001 |
| CHL | 27.73 | 1.553 | 916 | 17.849 | < 0.0001 |
| ANH | 30.65 | 1.465 | 916 | 20.928 | < 0.0001 |
| ***Communicator^TM^*** |  |  |  |  |  |
| CNH | 22.35 | 1.613 | 916 | 13.859 | < 0.0001 |
| CHL | 28.30 | 1.540 | 916 | 18.377 | < 0.0001 |
| ANH | 31.22 | 1.462 | 916 | 21.352 | < 0.0001 |
| ***Hospital*** |  |  |  |  |  |
| CNH | 1.36 | 1.618 | 916 | 0.843 | 0.3994 |
| CHL | 7.31 | 1.547 | 916 | 4.726 | < 0.0001 |
| ANH | 10.23 | 1.459 | 916 | 7.015 | < 0.0001 |
| ***Fabric*** |  |  |  |  |  |
| CNH | 1.83 | 1.621 | 916 | 1.129 | 0.2592 |
| CHL | 7.78 | 1.550 | 916 | 5.016 | < 0.0001 |
| ANH | 10.70 | 1.467 | 916 | 7.293 | < 0.0001 |
| **Effect of Modality (per Mask and Group)** | |  |  |  |  |
| ***CNH*** |  |  |  |  |  |
| No Mask | 20.86 | 1.657 | 916 | 12.590 | < 0.0001 |
| ClearMask^TM^ | 21.78 | 1.621 | 916 | 13.437 | < 0.0001 |
| Communicator^TM^ | 22.35 | 1.613 | 916 | 13.859 | < 0.0001 |
| Hospital | 1.36 | 1.618 | 916 | 0.843 | 0.3994 |
| Fabric | 1.83 | 1.621 | 916 | 1.129 | 0.2592 |
| ***CHL*** |  |  |  |  |  |
| No Mask | 26.81 | 1.585 | 916 | 16.920 | < 0.0001 |
| ClearMask^TM^ | 27.73 | 1.553 | 916 | 17.849 | < 0.0001 |
| Communicator^TM^ | 28.30 | 1.540 | 916 | 18.377 | < 0.0001 |
| Hospital | 7.31 | 1.547 | 916 | 4.726 | < 0.0001 |
| Fabric | 7.78 | 1.550 | 916 | 5.016 | < 0.0001 |
| ***ANH*** |  |  |  |  |  |
| No Mask | 29.73 | 1.488 | 917 | 19.982 | < 0.0001 |
| ClearMask^TM^ | 30.65 | 1.465 | 916 | 20.928 | < 0.0001 |
| Communicator^TM^ | 31.22 | 1.462 | 916 | 21.352 | < 0.0001 |
| Hospital | 10.23 | 1.459 | 916 | 7.015 | < 0.0001 |
| Fabric | 10.70 | 1.467 | 916 | 7.293 | < 0.0001 |

| Supplemental Table 2. Linear model estimates and Post Hoc comparisons for consonant recognition accuracy in Experiment 2. Reference conditions are on the left. | | | | | |
| --- | --- | --- | --- | --- | --- |
|  | **Estimate** | **S. E.** | **df** | **t-value** | **p-value** |
| **SNR x Modality Interaction** |  |  |  |  |  |
|  | 11.98 | 1.760 | 372 | 6.808 | < 0.0001 |
| **Effect of SNR (per Modality and Mask)** | |  |  |  |  |
| AO (no mask) | -34.29 | 2.277 | 386 | -15.061 | < 0.0001 |
| AV (no mask) | -22.31 | 2.279 | 386 | -9.790 | < 0.0001 |
| AO (ClearMask^TM^) | -44.38 | 2.274 | 386 | -19.517 | < 0.0001 |
| AV (ClearMask^TM^) | -32.39 | 2.268 | 386 | -14.283 | < 0.0001 |
| AO (Communicator^TM^) | -41.55 | 2.260 | 386 | -18.385 | < 0.0001 |
| AV (Communicator^TM^) | -29.57 | 2.270 | 386 | -13.025 | < 0.0001 |
| AO (hospital) | -42.24 | 2.277 | 386 | -18.549 | < 0.0001 |
| AV (hospital) | -30.25 | 2.279 | 386 | -13.274 | < 0.0001 |
| AO (fabric) | -54.13 | 2.277 | 386 | -23.771 | < 0.0001 |
| AV (fabric) | -42.14 | 2.279 | 386 | -18.491 | < 0.0001 |
| **Effect of Modality (per SNR and Mask)** | |  |  |  |  |
| 0 dB (no mask) | 20.09 | 2.160 | 372 | 9.301 | < 0.0001 |
| -10 dB (no mask) | 32.07 | 2.160 | 372 | 14.848 | < 0.0001 |
| 0 dB (ClearMask^TM^) | 21.03 | 2.145 | 372 | 9.804 | < 0.0001 |
| -10 dB (ClearMask^TM^) | 33.01 | 2.154 | 372 | 15.330 | < 0.0001 |
| 0 dB (Communicator^TM^) | 23.49 | 2.153 | 372 | 10.910 | < 0.0001 |
| -10 dB (Communicator^TM^) | 35.48 | 2.145 | 372 | 16.540 | < 0.0001 |
| 0 dB (hospital) | 2.58 | 2.160 | 372 | 1.196 | 0.2323 |
| -10 dB (hospital) | 14.57 | 2.160 | 372 | 6.744 | < 0.0001 |
| 0 dB (fabric) | 4.34 | 2.160 | 372 | 2.008 | 0.0454 |
| -10 dB (fabric) | 16.32 | 2.160 | 372 | 7.556 | < 0.0001 |
| **SNR x Mask Interaction** |  |  |  |  |  |
| NoMask - ClearMask^TM^ | -10.08 | 2.781 | 372 | -3.625 | 0.0003 |
| No Mask - Communicator^TM^ | -7.26 | 2.781 | 372 | -2.610 | 0.0094 |
| NoMask - Hospital | -7.94 | 2.789 | 372 | -2.847 | 0.0047 |
| NoMask - Fabric | -19.83 | 2.789 | 372 | -7.110 | < 0.0001 |
| ClearMask^TM^ - Communicator^TM^ | 2.82 | 2.773 | 372 | 1.018 | 0.3095 |
| ClearMask^TM^ - Hospital | 2.14 | 2.781 | 372 | 0.769 | 0.4423 |
| ClearMask^TM^ - Fabric | -9.75 | 2.781 | 372 | -3.506 | 0.0005 |
| Communicator^TM^ - Hospital | -0.68 | 2.781 | 372 | -0.246 | 0.8061 |
| Communicator^TM^ - Fabric | -12.57 | 2.781 | 372 | -4.521 | < 0.0001 |
| Hospital - Fabric | 11.89 | 2.789 | 372 | 4.262 | < 0.0001 |
| **Mask x Modality Interaction** |  |  |  |  |  |
| No Mask - ClearMask^TM^ | 0.94 | 2.781 | 372 | 0.339 | 0.7346 |
| No Mask - Communicator^TM^ | 3.41 | 2.781 | 372 | 1.225 | 0.2214 |
| No Mask - Hospital | -17.50 | 2.789 | 372 | -6.275 | < 0.0001 |
| No Mask - Fabric | -15.75 | 2.789 | 372 | -5.647 | < 0.0001 |
| ClearMask^TM^ - Communicator^TM^ | 2.46 | 2.773 | 372 | 0.888 | 0.3750 |
| ClearMask^TM^ - Hospital | -18.45 | 2.781 | 372 | -6.632 | < 0.0001 |
| ClearMask^TM^ - Fabric | -16.69 | 2.781 | 372 | -6.003 | < 0.0001 |
| Communicator^TM^ - Hospital | -20.91 | 2.781 | 372 | -7.518 | < 0.0001 |
| Communicator^TM^ - Fabric | -19.16 | 2.781 | 372 | -6.888 | < 0.0001 |
| Hospital - Fabric | 1.75 | 2.789 | 372 | 0.628 | 0.5303 |
| **Effect of Mask (per SNR and Modality)** | |  |  |  |  |
| **AO 0 dB SNR** |  |  |  |  |  |
| NoMask - ClearMask^TM^ | -6.76 | 2.413 | 372 | -2.803 | 0.0053 |
| NoMask - Communicator^TM^ | -10.81 | 2.413 | 372 | -4.479 | < 0.0001 |
| NoMask - Hospital | 1.19 | 2.416 | 372 | 0.491 | 0.6239 |
| NoMask - Fabric | -12.12 | 2.416 | 372 | -5.018 | < 0.0001 |
| ClearMask^TM^ - Communicator^TM^ | -4.05 | 2.411 | 372 | -1.678 | 0.0942 |
| ClearMask^TM^ - Hospital | 7.95 | 2.413 | 372 | 3.294 | 0.0011 |
| ClearMask^TM^ - Fabric | -5.36 | 2.413 | 372 | -2.220 | 0.0270 |
| Communicator^TM^ - Hospital | 11.99 | 2.413 | 372 | 4.970 | < 0.0001 |
| Communicator^TM^ - Fabric | -1.31 | 2.413 | 372 | -0.544 | 0.5869 |
| Hospital - Fabric | 13.31 | 2.416 | 372 | 5.509 | < 0.0001 |
| **AO -10 dB SNR** |  |  |  |  |  |
| NoMask - ClearMask^TM^ | -16.85 | 2.413 | 372 | -6.980 | < 0.0001 |
| NoMask - Communicator^TM^ | -18.07 | 2.394 | 372 | -7.546 | < 0.0001 |
| NoMask - Hospital | -6.76 | 2.416 | 372 | -2.797 | 0.0054 |
| NoMask - Fabric | -31.95 | 2.416 | 372 | -13.227 | < 0.0001 |
| ClearMask^TM^ - Communicator^TM^ | -1.22 | 2.392 | 372 | -0.511 | 0.6094 |
| ClearMask^TM^ - Hospital | 10.09 | 2.413 | 372 | 4.180 | < 0.0001 |
| ClearMask^TM^ - Fabric | -15.11 | 2.413 | 372 | -6.260 | < 0.0001 |
| Communicator^TM^ - Hospital | -20.91 | 2.781 | 372 | -7.518 | < 0.0001 |
| Communicator^TM^ - Fabric | -19.16 | 2.781 | 372 | -6.888 | < 0.0001 |
| Hospital - Fabric | -25.20 | 2.416 | 372 | -10.430 | < 0.0001 |
| **AV 0 dB SNR** |  |  |  |  |  |
| NoMask - ClearMask^TM^ | -5.82 | 2.395 | 372 | -2.431 | 0.0155 |
| NoMask - Communicator^TM^ | -7.40 | 2.413 | 372 | -3.067 | 0.0023 |
| NoMask - Hospital | -16.32 | 2.416 | 372 | -6.755 | < 0.0001 |
| NoMask - Fabric | -27.87 | 2.416 | 372 | -11.538 | < 0.0001 |
| ClearMask^TM^ - Communicator^TM^ | -1.58 | 2.392 | 372 | -0.661 | 0.5088 |
| ClearMask^TM^ - Hospital | -10.50 | 2.395 | 372 | -4.384 | < 0.0001 |
| ClearMask^TM^ - Fabric | -22.05 | 2.395 | 372 | -9.209 | < 0.0001 |
| Communicator^TM^ - Hospital | -8.92 | 2.413 | 372 | -3.694 | 0.0003 |
| Communicator^TM^ - Fabric | -20.47 | 2.413 | 372 | -8.482 | < 0.0001 |
| Hospital - Fabric | -11.55 | 2.416 | 372 | -4.783 | < 0.0001 |
| **AV -10 dB SNR** |  |  |  |  |  |
| NoMask - ClearMask^TM^ | -15.90 | 2.413 | 372 | -6.589 | < 0.0001 |
| NoMask - Communicator^TM^ | -14.66 | 2.413 | 372 | -6.075 | < 0.0001 |
| NoMask - Hospital | -24.26 | 2.416 | 372 | -10.043 | < 0.0001 |
| NoMask - Fabric | -47.70 | 2.416 | 372 | -19.748 | < 0.0001 |
| ClearMask^TM^ - Communicator^TM^ | 1.24 | 2.411 | 372 | 0.514 | 0.6073 |
| ClearMask^TM^ - Hospital | -8.36 | 2.413 | 372 | -3.463 | 0.0006 |
| ClearMask^TM^ - Fabric | -31.80 | 2.413 | 372 | -13.178 | < 0.0001 |
| Communicator^TM^ - Hospital | -9.60 | 2.413 | 372 | -3.977 | 0.0001 |
| Communicator^TM^ - Fabric | -33.04 | 2.413 | 372 | -13.692 | < 0.0001 |
| Hospital - Fabric | -23.44 | 2.416 | 372 | -9.705 | < 0.0001 |

| Supplemental Table 3. Linear model estimates and Post Hoc comparisons for comparison between CHL tested remotely at 0 dB SNR and ANH tested in the lab at -10 dB SNR. Reference conditions are on the left. | | | | | |
| --- | --- | --- | --- | --- | --- |
|  | **Estimate** | **S. E.** | **df** | **t-value** | **p-value** |
| **Group x Modality Interaction** |  |  |  |  |  |
| CHL - ANH : AO - AV | 6.62 | 1.809 | 471 | 3.661 | 0.0003 |
| **Group x Mask Interaction** |  |  |  |  |  |
| NoMask - ClearMask^TM^ | -5.39 | 2.882 | 471 | -1.869 | 0.0622 |
| NoMask - Communicator^TM^ | -3.80 | 2.861 | 471 | -1.328 | 0.1850 |
| NoMask - Hospital | 0.27 | 2.877 | 471 | 0.093 | 0.9256 |
| NoMask - Fabric | -13.91 | 2.877 | 471 | -4.833 | < 0.0001 |
| ClearMask^TM^ - Communicator^TM^ | 1.59 | 2.842 | 471 | 0.559 | 0.5767 |
| ClearMask^TM^ - Hospital | 5.65 | 2.860 | 471 | 1.977 | 0.0486 |
| ClearMask^TM^ - Fabric | -8.52 | 2.860 | 471 | -2.979 | 0.0030 |
| Communicator^TM^ - Hospital | 4.07 | 2.838 | 471 | 1.433 | 0.1526 |
| Communicator^TM^ - Fabric | -10.11 | 2.838 | 471 | -3.561 | 0.0004 |
| Hospital - Fabric | -14.17 | 2.856 | 471 | -4.963 | < 0.0001 |
| **Effect of Group (per Modality and Mask)** | |  |  |  |  |
| AO (no mask) | -6.23 | 4.482 | 41 | -1.389 | 0.1724 |
| AV (no mask) | 0.40 | 4.491 | 41 | 0.088 | 0.9301 |
| AO (ClearMask^TM^) | -11.61 | 4.477 | 41 | -2.594 | 0.0131 |
| AV (ClearMask^TM^) | -4.99 | 4.476 | 41 | -1.115 | 0.2715 |
| AO (Communicator^TM^) | -10.02 | 4.457 | 40 | -2.249 | 0.0301 |
| AV (Communicator^TM^) | -3.40 | 4.468 | 41 | -0.761 | 0.4508 |
| AO (hospital) | -5.96 | 4.475 | 41 | -1.331 | 0.1906 |
| AV (hospital) | 0.66 | 4.473 | 41 | 0.149 | 0.8826 |
| AO (fabric) | -20.13 | 4.473 | 41 | -4.501 | 0.0001 |
| AV (fabric) | -13.51 | 4.475 | 41 | -3.019 | 0.0044 |
| **Effect of Modality (per Group and Mask)** | |  |  |  |  |
| CHL (no mask) | 30.03 | 2.144 | 471 | 14.005 | < 0.0001 |
| ANH (no mask) | 36.65 | 2.282 | 471 | 16.065 | < 0.0001 |
| CHL (ClearMask^TM^) | 28.00 | 2.101 | 471 | 13.326 | < 0.0001 |
| ANH (ClearMask^TM^) | 34.62 | 2.263 | 471 | 15.300 | < 0.0001 |
| CHL (Communicator^TM^) | 27.92 | 2.067 | 471 | 13.508 | < 0.0001 |
| ANH (Communicator^TM^) | 34.54 | 2.237 | 471 | 15.442 | < 0.0001 |
| CHL (hospital) | 4.24 | 2.091 | 471 | 2.028 | 0.0432 |
| ANH (hospital) | 10.86 | 2.258 | 471 | 4.809 | < 0.0001 |
| CHL (fabric) | 8.29 | 2.090 | 471 | 3.968 | 0.0001 |
| ANH (fabric) | 14.92 | 2.258 | 471 | 6.605 | < 0.0001 |

| Supplemental Table 4. Linear model estimates and Post Hoc comparisons for analysis of auditory-only phonetic feature transmission in ANH tested at -10 dB SNR. Reference conditions are on the left. | | | | | |
| --- | --- | --- | --- | --- | --- |
|  | **Estimate** | **S. E.** | **df** | **t-value** | **p-value** |
| **Effect of Feature (per Mask Condition)** |  |  |  |  |  |
| ***No Mask*** |  |  |  |  |  |
| Place - Manner | 5.47 | 2.742 | 279 | 1.993 | 0.0473 |
| Place - Voicing | 16.50 | 2.742 | 279 | 6.016 | < 0.0001 |
| Manner - Voicing | 11.03 | 2.742 | 279 | 4.023 | 0.0001 |
| ***ClearMask^TM^*** |  |  |  |  |  |
| Place - Manner | 9.94 | 3.878 | 279 | 2.563 | 0.0109 |
| Place - Voicing | 10.27 | 3.878 | 279 | 2.648 | 0.0086 |
| Manner - Voicing | 0.33 | 3.878 | 279 | 0.085 | 0.9320 |
| ***Communicator^TM^*** |  |  |  |  |  |
| Place - Manner | 5.45 | 3.832 | 279 | 1.422 | 0.1562 |
| Place - Voicing | 7.06 | 3.832 | 279 | 1.842 | 0.0666 |
| Manner - Voicing | -1.61 | 3.832 | 279 | -0.420 | 0.6748 |
| ***Hospital*** |  |  |  |  |  |
| Place - Manner | 3.82 | 3.878 | 279 | 0.986 | 0.3250 |
| Place - Voicing | 5.92 | 3.878 | 279 | 1.527 | 0.1279 |
| Manner - Voicing | 2.10 | 3.878 | 279 | 0.541 | 0.5889 |
| ***Fabric*** |  |  |  |  |  |
| Place - Manner | 8.33 | 3.878 | 279 | 2.149 | 0.0325 |
| Place - Voicing | 14.82 | 3.878 | 279 | 3.822 | 0.0002 |
| Manner - Voicing | 6.49 | 3.878 | 279 | 1.673 | 0.0954 |
| **Effect of Mask (re: No Mask, per Feature)** | |  |  |  |  |
| ***ClearMask^TM^*** |  |  |  |  |  |
| Place | -16.51 | 2.742 | 279 | -6.019 | < 0.0001 |
| Manner | -6.57 | 2.742 | 279 | -2.394 | 0.0173 |
| Voicing | -6.24 | 2.742 | 279 | -2.274 | 0.0237 |
| ***Communicator^TM^*** |  |  |  |  |  |
| Place | -14.45 | 2.710 | 279 | -5.330 | < 0.0001 |
| Manner | -7.39 | 2.710 | 279 | -2.726 | 0.0068 |
| Voicing | -9.00 | 2.710 | 279 | -3.320 | 0.0010 |
| ***Hospital*** |  |  |  |  |  |
| Place | -5.91 | 2.742 | 279 | -2.156 | 0.0320 |
| Manner | -2.09 | 2.742 | 279 | -0.761 | 0.4471 |
| Voicing | 0.01 | 2.742 | 279 | 0.004 | 0.9970 |
| ***Fabric*** |  |  |  |  |  |
| Place | -27.49 | 2.742 | 279 | -10.025 | < 0.0001 |
| Manner | -19.16 | 2.742 | 279 | -6.986 | < 0.0001 |
| Voicing | -12.67 | 2.742 | 279 | -4.620 | < 0.0001 |
| **Effect of Mask (per Feature)** |  |  |  |  |  |
| ***Place*** |  |  |  |  |  |
| No Mask - ClearMask^TM^ | -16.51 | 2.742 | 279 | -6.019 | < 0.0001 |
| No Mask - Communicator^TM^ | -14.45 | 2.710 | 279 | -5.330 | < 0.0001 |
| No Mask - Hospital | -5.91 | 2.742 | 279 | -2.156 | 0.0320 |
| No Mask - Fabric | -27.49 | 2.742 | 279 | -10.025 | < 0.0001 |
| ClearMask^TM^ - Communicator^TM^ | 2.06 | 2.710 | 279 | 0.760 | 0.4477 |
| ClearMask^TM^ - Hospital | 10.59 | 2.742 | 279 | 3.863 | 0.0001 |
| ClearMask^TM^ - Fabric | -10.99 | 2.742 | 279 | -4.006 | 0.0001 |
| Communicator^TM^ - Hospital | 8.53 | 2.710 | 279 | 3.149 | 0.0018 |
| Communicator^TM^ - Fabric | -13.05 | 2.710 | 279 | -4.814 | < 0.0001 |
| Hospital - Fabric | -21.58 | 2.742 | 279 | -7.869 | < 0.0001 |
| ***Manner*** |  |  |  |  |  |
| No Mask - ClearMask^TM^ | -6.57 | 2.742 | 279 | -2.394 | 0.0173 |
| No Mask - Communicator^TM^ | -7.39 | 2.710 | 279 | -2.726 | 0.0068 |
| No Mask - Hospital | -2.09 | 2.742 | 279 | -0.761 | 0.4471 |
| No Mask - Fabric | -19.16 | 2.742 | 279 | -6.986 | < 0.0001 |
| ClearMask^TM^ - Communicator^TM^ | -0.82 | 2.710 | 279 | -0.303 | 0.7621 |
| ClearMask^TM^ - Hospital | 4.48 | 2.742 | 279 | 1.633 | 0.1036 |
| ClearMask^TM^ - Fabric | -12.59 | 2.742 | 279 | -4.592 | < 0.0001 |
| Communicator^TM^ - Hospital | 5.30 | 2.710 | 279 | 1.955 | 0.0515 |
| Communicator^TM^ - Fabric | -11.77 | 2.710 | 279 | -4.343 | < 0.0001 |
| Hospital - Fabric | -17.07 | 2.742 | 279 | -6.225 | < 0.0001 |
| ***Voicing*** |  |  |  |  |  |
| No Mask - ClearMask^TM^ | -6.24 | 2.742 | 279 | -2.274 | 0.0237 |
| No Mask - Communicator^TM^ | -9.00 | 2.710 | 279 | -3.320 | 0.0010 |
| No Mask - Hospital | 0.01 | 2.742 | 279 | 0.004 | 0.9970 |
| No Mask - Fabric | -12.67 | 2.742 | 279 | -4.620 | < 0.0001 |
| ClearMask^TM^ - Communicator^TM^ | -2.76 | 2.710 | 279 | -1.019 | 0.3091 |
| ClearMask^TM^ - Hospital | 6.25 | 2.742 | 279 | 2.277 | 0.0235 |
| ClearMask^TM^ - Fabric | -6.43 | 2.742 | 279 | -2.346 | 0.0197 |
| Communicator^TM^ - Hospital | 9.01 | 2.710 | 279 | 3.323 | 0.0010 |
| Communicator^TM^ - Fabric | -3.67 | 2.710 | 279 | -1.355 | 0.1765 |
| Hospital - Fabric | -12.68 | 2.742 | 279 | -4.624 | < 0.0001 |
| **Mask x Feature Interaction** |  |  |  |  |  |
| ***Place: Manner*** |  |  |  |  |  |
| No Mask - ClearMask^TM^ | 9.94 | 3.878 | 279 | 2.563 | 0.0109 |
| No Mask - Communicator^TM^ | 7.06 | 3.832 | 279 | 1.842 | 0.0666 |
| No Mask - Hospital | 3.82 | 3.878 | 279 | 0.986 | 0.3250 |
| No Mask - Fabric | 8.33 | 3.878 | 279 | 2.149 | 0.0325 |
| ClearMask^TM^ - Communicator^TM^ | -2.88 | 3.832 | 279 | -0.752 | 0.4526 |
| ClearMask^TM^ - Hospital | -6.12 | 3.878 | 279 | -1.577 | 0.1159 |
| ClearMask^TM^ - Fabric | -1.61 | 3.878 | 279 | -0.414 | 0.6791 |
| Communicator^TM^ - Hospital | -3.23 | 3.832 | 279 | -0.844 | 0.3994 |
| Communicator^TM^ - Fabric | 1.28 | 3.832 | 279 | 0.333 | 0.7394 |
| Hospital - Fabric | 4.51 | 3.878 | 279 | 1.163 | 0.2459 |
| ***Place: Voicing*** |  |  |  |  |  |
| No Mask - ClearMask^TM^ | 10.27 | 3.878 | 279 | 2.648 | 0.0086 |
| No Mask - Communicator^TM^ | 5.45 | 3.832 | 279 | 1.422 | 0.1562 |
| No Mask - Hospital | 5.92 | 3.878 | 279 | 1.527 | 0.1279 |
| No Mask - Fabric | 14.82 | 3.878 | 279 | 3.822 | 0.0002 |
| ClearMask^TM^ - Communicator^TM^ | -4.82 | 3.832 | 279 | -1.259 | 0.2093 |
| ClearMask^TM^ - Hospital | -4.35 | 3.878 | 279 | -1.121 | 0.2631 |
| ClearMask^TM^ - Fabric | 4.55 | 3.878 | 279 | 1.174 | 0.2415 |
| Communicator^TM^ - Hospital | 0.47 | 3.832 | 279 | 0.124 | 0.9017 |
| Communicator^TM^ - Fabric | 9.37 | 3.832 | 279 | 2.447 | 0.0150 |
| Hospital - Fabric | 8.90 | 3.878 | 279 | 2.295 | 0.0225 |
| ***Manner:Voicing*** |  |  |  |  |  |
| No Mask - ClearMask^TM^ | 0.33 | 3.878 | 279 | 0.085 | 0.9320 |
| No Mask - Communicator^TM^ | -1.61 | 3.832 | 279 | -0.420 | 0.6748 |
| No Mask - Hospital | 2.10 | 3.878 | 279 | 0.541 | 0.5889 |
| No Mask - Fabric | 6.49 | 3.878 | 279 | 1.673 | 0.0954 |
| ClearMask^TM^ - Communicator^TM^ | -1.94 | 3.832 | 279 | -0.506 | 0.6130 |
| ClearMask^TM^ - Hospital | 1.77 | 3.878 | 279 | 0.456 | 0.6490 |
| ClearMask^TM^ - Fabric | 6.16 | 3.878 | 279 | 1.588 | 0.1134 |
| Communicator^TM^ - Hospital | 3.71 | 3.832 | 279 | 0.968 | 0.3341 |
| Communicator^TM^ - Fabric | 8.10 | 3.832 | 279 | 2.114 | 0.0354 |
| Hospital - Fabric | 4.39 | 3.878 | 279 | 1.132 | 0.2585 |

| Supplemental Table 5. Linear model estimates and Post Hoc comparisons for analysis of audiovisual phonetic feature transmission in CHL. Reference conditions are on the left. | | | | | |
| --- | --- | --- | --- | --- | --- |
|  | **Estimate** | **S. E.** | **df** | **t-value** | **p-value** |
| **Effect of Feature (per Mask Condition)** | |  |  |  |  |
| ***No Mask*** |  |  |  |  |  |
| Place - Manner | -11.63 | 3.011 | 427 | -3.862 | 0.0001 |
| Place - Voicing | 2.62 | 3.011 | 427 | 0.869 | 0.3851 |
| Manner - Voicing | 14.25 | 3.011 | 427 | 4.731 | < 0.0001 |
| ***ClearMask^TM^*** |  |  |  |  |  |
| Place - Manner | -12.81 | 2.862 | 427 | -4.476 | < 0.0001 |
| Place - Voicing | 5.53 | 2.862 | 427 | 1.932 | 0.0540 |
| Manner - Voicing | 18.34 | 2.862 | 427 | 6.408 | < 0.0001 |
| ***Communicator^TM^*** |  |  |  |  |  |
| Place - Manner | -8.94 | 2.862 | 427 | -3.123 | 0.0019 |
| Place - Voicing | 8.03 | 2.862 | 427 | 2.806 | 0.0052 |
| Manner - Voicing | 16.97 | 2.862 | 427 | 5.929 | < 0.0001 |
| ***Hospital*** |  |  |  |  |  |
| Place - Manner | 4.71 | 2.817 | 427 | 1.671 | 0.0954 |
| Place - Voicing | 22.28 | 2.817 | 427 | 7.911 | < 0.0001 |
| Manner - Voicing | 17.58 | 2.817 | 427 | 6.240 | < 0.0001 |
| ***Fabric*** |  |  |  |  |  |
| Place - Manner | 5.41 | 2.862 | 427 | 1.892 | 0.0592 |
| Place - Voicing | 26.75 | 2.862 | 427 | 9.349 | < 0.0001 |
| Manner - Voicing | 21.34 | 2.862 | 427 | 7.457 | < 0.0001 |
| **Effect of Mask (re: No Mask, per Feature)** | |  |  |  |  |
| ***ClearMask^TM^*** |  |  |  |  |  |
| Place | -8.33 | 2.946 | 427 | -2.826 | 0.0049 |
| Manner | -9.51 | 2.946 | 427 | -3.227 | 0.0013 |
| Voicing | -5.42 | 2.946 | 427 | -1.838 | 0.0668 |
| ***Communicator^TM^*** |  |  |  |  |  |
| Place | -13.41 | 2.946 | 427 | -4.552 | < 0.0001 |
| Manner | -10.72 | 2.946 | 427 | -3.639 | 0.0003 |
| Voicing | -8.00 | 2.946 | 427 | -2.715 | 0.0069 |
| ***Hospital*** |  |  |  |  |  |
| Place | -32.82 | 2.925 | 427 | -11.222 | < 0.0001 |
| Manner | -16.48 | 2.925 | 427 | -5.636 | < 0.0001 |
| Voicing | -13.15 | 2.925 | 427 | -4.497 | < 0.0001 |
| ***Fabric*** |  |  |  |  |  |
| Place | -39.16 | 2.946 | 427 | -13.291 | < 0.0001 |
| Manner | -22.12 | 2.946 | 427 | -7.507 | < 0.0001 |
| Voicing | -15.02 | 2.946 | 427 | -5.099 | < 0.0001 |
| **Effect of Mask (per Feature)** |  |  |  |  |  |
| ***Place*** |  |  |  |  |  |
| No Mask - ClearMask^TM^ | -8.33 | 2.946 | 427 | -2.826 | 0.0049 |
| No Mask - Communicator^TM^ | -13.41 | 2.946 | 427 | -4.552 | < 0.0001 |
| No Mask - Hospital | -32.82 | 2.925 | 427 | -11.222 | < 0.0001 |
| No Mask - Fabric | -39.16 | 2.946 | 427 | -13.291 | < 0.0001 |
| ClearMask^TM^ - Communicator^TM^ | -5.08 | 2.862 | 427 | -1.777 | 0.0763 |
| ClearMask^TM^ - Hospital | -24.49 | 2.840 | 427 | -8.624 | < 0.0001 |
| ClearMask^TM^ - Fabric | -30.83 | 2.862 | 427 | -10.774 | < 0.0001 |
| Communicator^TM^ - Hospital | -19.41 | 2.840 | 427 | -6.834 | < 0.0001 |
| Communicator^TM^ - Fabric | -25.75 | 2.862 | 427 | -8.997 | < 0.0001 |
| Hospital - Fabric | -6.34 | 2.840 | 427 | -2.232 | 0.0261 |
| ***Manner*** |  |  |  |  |  |
| No Mask - ClearMask^TM^ | -9.51 | 2.946 | 427 | -3.227 | 0.0013 |
| No Mask - Communicator^TM^ | -10.72 | 2.946 | 427 | -3.639 | 0.0003 |
| No Mask - Hospital | -16.48 | 2.925 | 427 | -5.636 | < 0.0001 |
| No Mask - Fabric | -22.12 | 2.946 | 427 | -7.507 | < 0.0001 |
| ClearMask^TM^ - Communicator^TM^ | -1.21 | 2.862 | 427 | -0.424 | 0.6716 |
| ClearMask^TM^ - Hospital | -6.98 | 2.840 | 427 | -2.456 | 0.0144 |
| ClearMask^TM^ - Fabric | -12.61 | 2.862 | 427 | -4.407 | < 0.0001 |
| Communicator^TM^ - Hospital | -5.76 | 2.840 | 427 | -2.029 | 0.0431 |
| Communicator^TM^ - Fabric | -11.40 | 2.862 | 427 | -3.982 | 0.0001 |
| Hospital - Fabric | -5.63 | 2.840 | 427 | -1.984 | 0.0479 |
| ***Voicing*** |  |  |  |  |  |
| No Mask - ClearMask^TM^ | -5.42 | 2.946 | 427 | -1.838 | 0.0668 |
| No Mask - Communicator^TM^ | -8.00 | 2.946 | 427 | -2.715 | 0.0069 |
| No Mask - Hospital | -13.15 | 2.925 | 427 | -4.497 | < 0.0001 |
| No Mask - Fabric | -15.02 | 2.946 | 427 | -5.099 | < 0.0001 |
| ClearMask^TM^ - Communicator^TM^ | -2.58 | 2.862 | 427 | -0.903 | 0.3671 |
| ClearMask^TM^ - Hospital | -7.74 | 2.840 | 427 | -2.725 | 0.0067 |
| ClearMask^TM^ - Fabric | -9.61 | 2.862 | 427 | -3.358 | 0.0009 |
| Communicator^TM^ - Hospital | -5.15 | 2.840 | 427 | -1.815 | 0.0702 |
| Communicator^TM^ - Fabric | -7.03 | 2.862 | 427 | -2.455 | 0.0145 |
| Hospital - Fabric | -1.87 | 2.840 | 427 | -0.659 | 0.5105 |
| **Mask x Feature Interaction** |  |  |  |  |  |
| ***Place: Manner*** |  |  |  |  |  |
| No Mask - ClearMask^TM^ | -1.18 | 4.154 | 427 | -0.284 | 0.7764 |
| No Mask - Communicator^TM^ | 2.69 | 4.154 | 427 | 0.648 | 0.5176 |
| No Mask - Hospital | 16.34 | 4.123 | 427 | 3.962 | 0.0001 |
| No Mask - Fabric | 17.04 | 4.154 | 427 | 4.102 | < 0.0001 |
| ClearMask^TM^ - Communicator^TM^ | 3.87 | 4.047 | 427 | 0.956 | 0.3394 |
| ClearMask^TM^ - Hospital | 17.52 | 4.016 | 427 | 4.362 | < 0.0001 |
| ClearMask^TM^ - Fabric | 18.22 | 4.047 | 427 | 4.502 | < 0.0001 |
| Communicator^TM^ - Hospital | 13.65 | 4.016 | 427 | 3.398 | 0.0007 |
| Communicator^TM^ - Fabric | 14.35 | 4.047 | 427 | 3.546 | 0.0004 |
| Hospital - Fabric | 0.71 | 4.016 | 427 | 0.176 | 0.8606 |
| ***Place: Voicing*** |  |  |  |  |  |
| No Mask - ClearMask^TM^ | 2.91 | 4.154 | 427 | 0.701 | 0.4837 |
| No Mask - Communicator^TM^ | 5.41 | 4.154 | 427 | 1.303 | 0.1933 |
| No Mask - Hospital | 19.67 | 4.123 | 427 | 4.770 | < 0.0001 |
| No Mask - Fabric | 24.14 | 4.154 | 427 | 5.810 | < 0.0001 |
| ClearMask^TM^ - Communicator^TM^ | 2.50 | 4.047 | 427 | 0.618 | 0.5370 |
| ClearMask^TM^ - Hospital | 16.76 | 4.016 | 427 | 4.172 | < 0.0001 |
| ClearMask^TM^ - Fabric | 21.22 | 4.047 | 427 | 5.244 | < 0.0001 |
| Communicator^TM^ - Hospital | 14.25 | 4.016 | 427 | 3.550 | 0.0004 |
| Communicator^TM^ - Fabric | 18.72 | 4.047 | 427 | 4.626 | < 0.0001 |
| Hospital - Fabric | 4.47 | 4.016 | 427 | 1.113 | 0.2663 |
| ***Manner:Voicing*** |  |  |  |  |  |
| No Mask - ClearMask^TM^ | 4.09 | 4.154 | 427 | 0.985 | 0.3252 |
| No Mask - Communicator^TM^ | 2.72 | 4.154 | 427 | 0.655 | 0.5127 |
| No Mask - Hospital | 3.33 | 4.123 | 427 | 0.808 | 0.4198 |
| No Mask - Fabric | 7.09 | 4.154 | 427 | 1.708 | 0.0884 |
| ClearMask^TM^ - Communicator^TM^ | -1.37 | 4.047 | 427 | -0.339 | 0.7351 |
| ClearMask^TM^ - Hospital | -0.76 | 4.016 | 427 | -0.190 | 0.8495 |
| ClearMask^TM^ - Fabric | 3.00 | 4.047 | 427 | 0.742 | 0.4587 |
| Communicator^TM^ - Hospital | 0.61 | 4.016 | 427 | 0.151 | 0.8797 |
| Communicator^TM^ - Fabric | 4.37 | 4.047 | 427 | 1.080 | 0.2807 |
| Hospital - Fabric | 3.76 | 4.016 | 427 | 0.937 | 0.3491 |

| Supplemental Table 6. Linear model estimates and Post Hoc comparisons for analysis of AV phonetic feature transmission in ANH tested at -10 dB SNR. | | | | | |
| --- | --- | --- | --- | --- | --- |
|  | **Estimate** | **S. E.** | **df** | **t-value** | **p-value** |
| **Effect of Feature (per Mask Condition)** | |  |  |  |  |
| ***No Mask*** |  |  |  |  |  |
| Place:Manner | -15.76 | 2.879 | 276 | -5.474 | <0.0001 |
| Place:Voicing | -5.32 | 2.879 | 276 | -1.848 | 0.0656 |
| Manner:Voicing | 10.44 | 2.879 | 276 | 3.625 | 0.0003 |
| **ClearMask^TM^** |  |  |  |  |  |
| Place:Manner | -16.36 | 2.879 | 276 | -5.685 | <0.0001 |
| Place:Voicing | -9.97 | 2.879 | 276 | -3.463 | 0.0006 |
| Manner:Voicing | 6.39 | 2.879 | 276 | 2.221 | 0.0271 |
| ***Communicator*^TM^** |  |  |  |  |  |
| Place:Manner | -19.57 | 2.879 | 276 | -6.799 | <0.0001 |
| Place:Voicing | -12.82 | 2.879 | 276 | -4.455 | <0.0001 |
| Manner:Voicing | 6.75 | 2.879 | 276 | 2.344 | 0.0198 |
| ***Hospital*** |  |  |  |  |  |
| Place:Manner | -2.99 | 2.879 | 276 | -1.040 | 0.2992 |
| Place:Voicing | 10.49 | 2.879 | 276 | 3.646 | 0.0003 |
| Manner:Voicing | 13.49 | 2.879 | 276 | 4.686 | <0.0001 |
| ***Fabric*** |  |  |  |  |  |
| Place:Manner | -1.54 | 2.879 | 276 | -0.536 | 0.5926 |
| Place:Voicing | 16.85 | 2.879 | 276 | 5.855 | <0.0001 |
| Manner:Voicing | 18.39 | 2.879 | 276 | 6.390 | <0.0001 |
| **Effect of Mask (re: No Mask, per Feature)** | |  |  |  |  |
| ***ClearMask^TM^*** |  |  |  |  |  |
| Place | -9.86 | 2.879 | 276 | -3.424 | 0.0007 |
| Manner | -10.46 | 2.879 | 276 | -3.635 | 0.0003 |
| Voicing | -14.51 | 2.879 | 276 | -5.039 | <0.0001 |
| ***Communicator^TM^*** |  |  |  |  |  |
| Place | -7.95 | 2.879 | 276 | -2.761 | 0.0062 |
| Manner | -11.76 | 2.879 | 276 | -4.086 | 0.0001 |
| Voicing | -15.45 | 2.879 | 276 | -5.367 | <0.0001 |
| ***Hospital*** |  |  |  |  |  |
| Place | -29.93 | 2.879 | 276 | -10.399 | <0.0001 |
| Manner | -17.17 | 2.879 | 276 | -5.965 | <0.0001 |
| Voicing | -14.12 | 2.879 | 276 | -4.905 | <0.0001 |
| ***Fabric*** |  |  |  |  |  |
| Place | -48.97 | 2.879 | 276 | -17.011 | <0.0001 |
| Manner | -34.75 | 2.879 | 276 | -12.072 | <0.0001 |
| Voicing | -26.79 | 2.879 | 276 | -9.308 | <0.0001 |
| **Effect of Mask (per Feature)** | |  |  |  |  |
| ***Place*** |  |  |  |  |  |
| No Mask - ClearMask^TM^ | -9.86 | 2.879 | 276 | -3.424 | 0.0007 |
| No Mask - Communicator^TM^ | -7.95 | 2.879 | 276 | -2.761 | 0.0062 |
| No Mask - Hospital | -29.93 | 2.879 | 276 | -10.399 | <0.0001 |
| No Mask - Fabric | -48.97 | 2.879 | 276 | -17.011 | <0.0001 |
| ClearMask^TM^ - Communicator^TM^ | 1.91 | 2.879 | 276 | 0.664 | 0.5073 |
| ClearMask^TM^ - Hospital | -20.08 | 2.879 | 276 | -6.975 | <0.0001 |
| ClearMask^TM^ - Fabric | -39.11 | 2.879 | 276 | -13.586 | <0.0001 |
| Communicator^TM^ - Hospital | -21.99 | 2.879 | 276 | -7.639 | <0.0001 |
| Communicator^TM^ - Fabric | -41.02 | 2.879 | 276 | -14.250 | <0.0001 |
| Hospital - Fabric | -19.03 | 2.879 | 276 | -6.612 | <0.0001 |
| ***Manner*** |  |  |  |  |  |
| No Mask - ClearMask^TM^ | -10.46 | 2.879 | 276 | -3.635 | 0.0003 |
| No Mask - Communicator^TM^ | -11.76 | 2.879 | 276 | -4.086 | 0.0001 |
| No Mask - Hospital | -17.17 | 2.879 | 276 | -5.965 | <0.0001 |
| No Mask - Fabric | -34.75 | 2.879 | 276 | -12.072 | <0.0001 |
| ClearMask^TM^ - Communicator^TM^ | -1.30 | 2.879 | 276 | -0.450 | 0.6527 |
| ClearMask^TM^ - Hospital | -6.71 | 2.879 | 276 | -2.330 | 0.0205 |
| ClearMask^TM^ - Fabric | -24.29 | 2.879 | 276 | -8.437 | <0.0001 |
| Communicator^TM^ - Hospital | -5.41 | 2.879 | 276 | -1.880 | 0.0612 |
| Communicator^TM^ - Fabric | -22.99 | 2.879 | 276 | -7.987 | <0.0001 |
| Hospital - Fabric | -17.58 | 2.879 | 276 | -6.107 | <0.0001 |
| ***Voicing*** |  |  |  |  |  |
| No Mask - ClearMask^TM^ | -14.51 | 2.879 | 276 | -5.039 | <0.0001 |
| No Mask - Communicator^TM^ | -15.45 | 2.879 | 276 | -5.367 | <0.0001 |
| No Mask - Hospital | -14.12 | 2.879 | 276 | -4.905 | <0.0001 |
| No Mask - Fabric | -26.79 | 2.879 | 276 | -9.308 | <0.0001 |
| ClearMask^TM^ - Communicator^TM^ | -0.94 | 2.879 | 276 | -0.328 | 0.7434 |
| ClearMask^TM^ - Hospital | 0.39 | 2.879 | 276 | 0.135 | 0.8931 |
| ClearMask^TM^ - Fabric | -12.29 | 2.879 | 276 | -4.268 | <0.0001 |
| Communicator^TM^ - Hospital | 1.33 | 2.879 | 276 | 0.462 | 0.6443 |
| Communicator^TM^ - Fabric | -11.34 | 2.879 | 276 | -3.940 | 0.0001 |
| Hospital - Fabric | -12.67 | 2.879 | 276 | -4.403 | <0.0001 |
| **Mask x Feature Interaction** | |  |  |  |  |
| ***Place: Manner*** |  |  |  |  |  |
| No Mask - ClearMask^TM^ | -0.61 | 4.071 | 276 | -0.149 | 0.8816 |
| No Mask - Communicator^TM^ | -3.81 | 4.071 | 276 | -0.937 | 0.3495 |
| No Mask - Hospital | 12.76 | 4.071 | 276 | 3.135 | 0.0019 |
| No Mask - Fabric | 14.21 | 4.071 | 276 | 3.492 | 0.0006 |
| ClearMask^TM^ - Communicator^TM^ | -3.21 | 4.071 | 276 | -0.788 | 0.4314 |
| ClearMask^TM^ - Hospital | 13.37 | 4.071 | 276 | 3.284 | 0.0012 |
| ClearMask^TM^ - Fabric | 14.82 | 4.071 | 276 | 3.641 | 0.0003 |
| Communicator^TM^ - Hospital | 16.58 | 4.071 | 276 | 4.072 | 0.0001 |
| Communicator^TM^ - Fabric | 18.03 | 4.071 | 276 | 4.429 | <0.0001 |
| Hospital - Fabric | 1.45 | 4.071 | 276 | 0.357 | 0.7216 |
| ***Place: Voicing*** | |  |  |  |  |
| No Mask - ClearMask^TM^ | -4.65 | 4.071 | 276 | -1.142 | 0.2545 |
| No Mask - Communicator^TM^ | -7.50 | 4.071 | 276 | -1.843 | 0.0664 |
| No Mask - Hospital | 15.82 | 4.071 | 276 | 3.885 | 0.0001 |
| No Mask - Fabric | 22.17 | 4.071 | 276 | 5.447 | <0.0001 |
| ClearMask^TM^ - Communicator^TM^ | -2.85 | 4.071 | 276 | -0.701 | 0.4838 |
| ClearMask^TM^ - Hospital | 20.46 | 4.071 | 276 | 5.027 | <0.0001 |
| ClearMask^TM^ - Fabric | 26.82 | 4.071 | 276 | 6.589 | <0.0001 |
| Communicator^TM^ - Hospital | 23.32 | 4.071 | 276 | 5.728 | <0.0001 |
| Communicator^TM^ - Fabric | 29.68 | 4.071 | 276 | 7.290 | <0.0001 |
| Hospital - Fabric | 6.36 | 4.071 | 276 | 1.562 | 0.1195 |
| ***Manner: Voicing*** | |  |  |  |  |
| No Mask - ClearMask^TM^ | -4.04 | 4.071 | 276 | -0.993 | 0.3216 |
| No Mask - Communicator^TM^ | -3.69 | 4.071 | 276 | -0.906 | 0.3657 |
| No Mask - Hospital | 3.05 | 4.071 | 276 | 0.750 | 0.4540 |
| No Mask - Fabric | 7.96 | 4.071 | 276 | 1.955 | 0.0516 |
| ClearMask^TM^ - Communicator^TM^ | 0.35 | 4.071 | 276 | 0.087 | 0.9309 |
| ClearMask^TM^ - Hospital | 7.09 | 4.071 | 276 | 1.743 | 0.0825 |
| ClearMask^TM^ - Fabric | 12.00 | 4.071 | 276 | 2.948 | 0.0035 |
| Communicator^TM^ - Hospital | 6.74 | 4.071 | 276 | 1.656 | 0.0989 |
| Communicator^TM^ - Fabric | 11.65 | 4.071 | 276 | 2.861 | 0.0045 |
| Hospital - Fabric | 4.91 | 4.071 | 276 | 1.205 | 0.2291 |

| Supplemental Table 7. Linear model estimates and Post Hoc comparisons for analysis of visual-only phonetic feature transmission in ANH, CNH, and CHL. Reference conditions are on the left. | | | | | |
| --- | --- | --- | --- | --- | --- |
|  | **Estimate** | **S. E.** | **df** | **t-value** | **p-value** |
| **Effect of Group (per Feature)** | |  |  |  |  |
| ***Place*** |  |  |  |  |  |
| CHL - ANH | 13.63 | 3.017 | 97 | 4.519 | < 0.0001 |
| CHL - CNH | -5.91 | 3.767 | 102 | -1.569 | 0.1198 |
| ANH - CNH | -19.54 | 3.175 | 103 | -6.155 | < 0.0001 |
| ***Manner*** |  |  |  |  |  |
| CHL - ANH | -2.64 | 3.767 | 102 | -0.700 | 0.4858 |
| CHL - CNH | 7.15 | 3.017 | 97 | 2.369 | 0.0198 |
| ANH - CNH | -9.78 | 3.175 | 103 | -3.081 | 0.0026 |
| ***Voicing*** |  |  |  |  |  |
| CHL - ANH | 3.19 | 3.017 | 97 | 1.056 | 0.2934 |
| CHL - CNH | -3.82 | 3.767 | 102 | -1.014 | 0.3128 |
| ANH - CNH | -7.01 | 3.175 | 103 | -2.207 | 0.0295 |
| **Effect of Feature (per Group)** | |  |  |  |  |
| ***CHL*** |  |  |  |  |  |
| Place - Manner | -22.45 | 2.413 | 269 | -9.303 | < 0.0001 |
| Place - Voicing | -13.51 | 2.413 | 269 | -5.599 | < 0.0001 |
| Manner - Voicing | 8.94 | 2.413 | 269 | 3.704 | 0.0003 |
| ***ANH*** |  |  |  |  |  |
| Place - Manner | -28.94 | 1.455 | 269 | -19.884 | < 0.0001 |
| Place - Voicing | -23.96 | 1.455 | 269 | -16.463 | < 0.0001 |
| Manner - Voicing | 4.98 | 1.455 | 269 | 3.421 | 0.0007 |
| ***CNH*** |  |  |  |  |  |
| Place - Manner | -19.18 | 2.644 | 269 | -7.254 | < 0.0001 |
| Place - Voicing | -11.42 | 2.644 | 269 | -4.321 | < 0.0001 |
| Manner - Voicing | 7.75 | 2.644 | 269 | 2.933 | 0.0036 |
| **Group x Feature Interaction** |  |  |  |  |  |
| ***Place: Manner*** |  |  |  |  |  |
| CHL - ANH | -6.49 | 2.818 | 269 | -2.302 | 0.0221 |
| CHL - CNH | 3.27 | 3.579 | 269 | 0.915 | 0.3613 |
| ANH - CNH | 9.76 | 3.018 | 269 | 3.234 | 0.0014 |
| ***Place: Voicing*** |  |  |  |  |  |
| CHL - ANH | -10.45 | 2.818 | 269 | -3.707 | 0.0003 |
| CHL - CNH | 2.09 | 3.579 | 269 | 0.583 | 0.5602 |
| ANH - CNH | 12.54 | 3.018 | 269 | 4.154 | < 0.0001 |
| ***Manner: Voicing*** |  |  |  |  |  |
| CHL - ANH | -3.96 | 2.818 | 269 | -1.406 | 0.1610 |
| CHL - CNH | -1.19 | 3.579 | 269 | -0.331 | 0.7408 |
| ANH - CNH | 2.78 | 3.018 | 269 | 0.920 | 0.3585 |
